# Subsistence strategies in traditional societies distinguish gut microbiomes

**DOI:** 10.1038/ncomms7505

**Published:** 2015-03-25

**Authors:** Alexandra J. Obregon-Tito, Raul Y. Tito, Jessica Metcalf, Krithivasan Sankaranarayanan, Jose C. Clemente, Luke K. Ursell, Zhenjiang Zech Xu, Will Van Treuren, Rob Knight, Patrick M. Gaffney, Paul Spicer, Paul Lawson, Luis Marin-Reyes, Omar Trujillo-Villarroel, Morris Foster, Emilio Guija-Poma, Luzmila Troncoso-Corzo, Christina Warinner, Andrew T. Ozga, Cecil M. Lewis

**Affiliations:** 1 Department of Anthropology, University of Oklahoma, Dale Hall Tower, 521 Norman, Oklahoma 73019, USA; 2 Universidad Científica del Sur, Lima 18, Perú; 3 City of Hope, NCI-designated Comprehensive Cancer Center, Duarte, California 91010, USA; 4 Department of Chemistry and Biochemistry, University of Colorado, Boulder, Colorado 80309, USA; 5 Icahn School of Medicine at Mount Sinai, New York, New York 10029, USA; 6 Departments of Pediatrics and Computer Science & Engineering University of California San Diego, La Jolla, CA 92093, USA; 7 Oklahoma Medical Research Foundation, Oklahoma City, Oklahoma 73104, USA; 8 Instituto Nacional de Salud, Lima 11, Perú; 9 Old Dominion University, Norfolk, Virginia 23529, USA

## Abstract

Recent studies suggest that gut microbiomes of urban-industrialized societies are different from those of traditional peoples. Here we examine the relationship between lifeways and gut microbiota through taxonomic and functional potential characterization of faecal samples from hunter-gatherer and traditional agriculturalist communities in Peru and an urban-industrialized community from the US. We find that in addition to taxonomic and metabolic differences between urban and traditional lifestyles, hunter-gatherers form a distinct sub-group among traditional peoples. As observed in previous studies, we find that *Treponema* are characteristic of traditional gut microbiomes. Moreover, through genome reconstruction (2.2–2.5 MB, coverage depth × 26–513) and functional potential characterization, we discover these *Treponema* are diverse, fall outside of pathogenic clades and are similar to *Treponema succinifaciens*, a known carbohydrate metabolizer in swine. Gut *Treponema* are found in non-human primates and all traditional peoples studied to date, suggesting they are symbionts lost in urban-industrialized societies.

## Abstract (web summary)

The gut microbiomes of urban-industrialized societies differ from those of traditional rural societies and hunter-gatherers. Here the authors perform a comparative analysis of available and new gut microbiome data to provide fresh insight into these differences.

## Graphical abstract

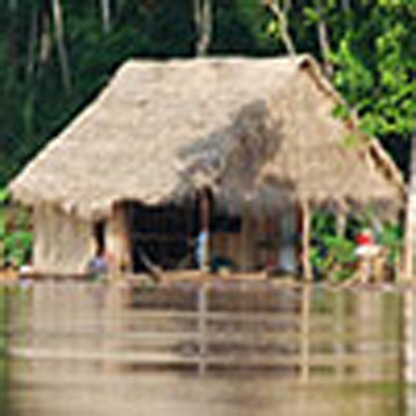

Understanding the human microbiome has the potential to transform health and medicine. Yet, despite large-scale sequencing efforts, the full extent of human gut microbial diversity remains underexplored. Extant people living traditional lifestyles are especially under-studied, limited to one population of hunter-gatherers from Tanzania^1^, and three rural agriculturalist communities in Burkina Faso^2^, Malawi and Venezuela^3^. Studies of peoples maintaining traditional subsistence practices are critical for understanding the ancestral state of the human microbiome and providing a foundation for understanding how the human microbiome responds to urbanism and Westernization, especially regarding diseases of civilization, such as obesity and chronic inflammatory disorders. To date, only two studies have focused on the gut microbiomes of communities exclusively eating local, non-industrially produced foods: a study by De Filippo *et al.*^2^ that focused on children up to 6 years old from Burkina Faso, whose diet was primarily composed of locally grown cereals, legumes and vegetables^2^, and a study by Schnorr *et al.*^1^ that explored the gut microbiome of African hunter-gatherers from Tanzania. A study on rural agriculturalist communities from Venezuela and Malawi^3^ included adults with more diverse diets including industrial goods such as soda in Malawi, and milk products, canned products and soda in Venezuela.

Because of their unique cultural, behavioural and ecological environment, we hypothesize that remote hunter-gatherer communities harbour novel microbiome profiles that depart from those previously described in urban and semi-urban settings, and that may be tailored to the specific dietary sources within each population. To test this hypothesis, here we use a combination of high throughput 16S ribosomal RNA (rRNA) gene amplicon sequencing and shotgun metagenomic sequencing to characterize the gut microbiota of peoples from three different lifeways: traditional hunter-gatherers, traditional agriculturalists and urban-industrialized peoples. In addition to previously published data, we provide novel data from: (1) the Matses, a remote hunter-gatherer population from the Peruvian Amazon; (2) Tunapuco, a traditional agricultural community from the Andean highlands; and (3) residents of Norman, Oklahoma, a typical US university community that serves as a comparative population following an urban-industrialized lifestyle.

## Results

### Diet and engagement

While both rural communities live within the national borders of Peru, the lifeways of the Matses and residents of Tunapuco are startlingly different. The Matses live at an elevation of 150 m above sea level in a pocket of natural hyperdiversity that extends across the Brazilian border, and, until recently, the Matses have been geographically, historically and socially, isolated^4^. The Matses are traditional hunter-gatherers whose subsistence focuses primarily on gathered tubers (*Manihot* spp.) and invasive plantains (*Musa* spp.) (Supplementary Table 1). Fish is their primary protein source, complemented by sporadic consumption of game meat (monkey, sloth, capybara, alligator and so on.). Consumption of dairy or processed food is very rare, and only as a result of sporadic visitors. In contrast, Tunapuco is situated in the central Andes, at an elevation between 2,500 and 3,100 m above sea level. The diet of this rural agriculturalist community is based on local agricultural produce and homegrown small animals. Their main sources of nutrition include stem tubers such as potatoes (*Solanum tuberosum* spp.) and root tubers like oca (*Oxalis tuberosa*) and mashua (*Tropaeolum tuberosum*), which they eat at every meal. Tocosh, a typical dish of the central Andes made out of potatoes that have been fermented in wet soil, is eaten at least once a week by families in Tunapuco (Supplementary Table 2). Residents of Tunapuco eat fruits that they buy from lowland rural communities from the same region. Guinea pig, pork, lamb and infrequent cow cheese are the main animal protein sources in their diet. Intake of dairy products and processed foods is limited, and rice and bread are the main products they buy to supplement their diet. Residents of Norman self-report diets typical of urban-industrial communities, with regular consumption of processed foods including canned fruits and vegetables, bread and prepackaged meals. In addition, residents of Norman also reported regular dairy consumption in the form of milk, cheese and other dairy products.

This study was conducted under the supervision of the University of Oklahoma and the Ethics Committee of the Peruvian National Institute of Health, in collaboration with the Matses and Tunapuco communities (Supplementary Fig. 1). Our model of research with indigenous populations consists of longitudinal engagement; Community Based Participatory Research was designed^5^ to ethically engage vulnerable indigenous communities in microbiome research (Methods). Our participants range from 1–52 years of age for the Matses, 3–63 years of age for Tunapuco and 7–50 years of age for the Norman population. Body mass index, age and sex of our participants are summarized in Supplementary Table 3.

### Rural communities have higher richness

**Figure 1:**
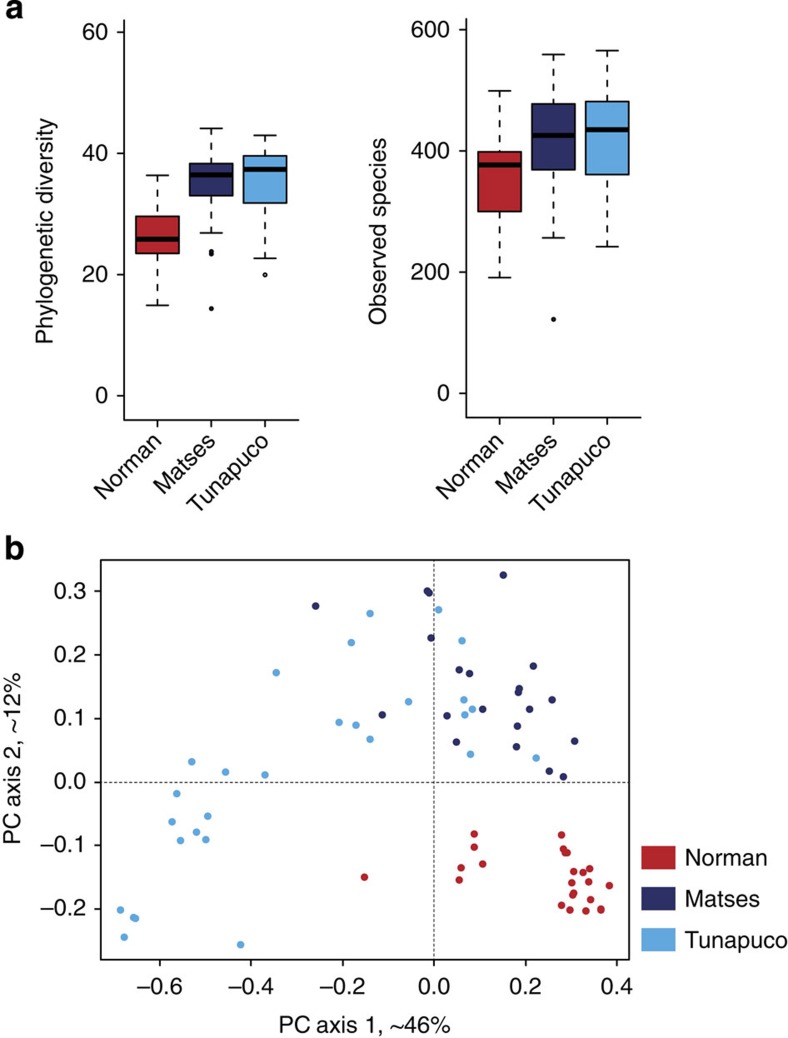
Alpha- and beta-diversity comparisons of the gut microbiomes of the Matses, Tunapuco and Norman populations. Analyses were performed on 16S rRNA V4 region data, with a rarefaction depth of 10,000 reads per sample. (**a**) Alpha diversity comparisons based on phylogenetic and non-phylogenetic richness (Faith’s PD, observed species). The urban population has significantly lower microbial richness compared with the two rural populations. This observation is robust and observable even with <5,000 reads per sample (Supplementary Fig. 2). Whiskers in the boxplot represent the range of minimum and maximum alpha diversity values within a population, excluding outliers (**b**) Principal coordinates analysis of weighted UniFrac distances. Proportion of variance explained by each principal coordinate axis is denoted in the corresponding axis label. The rural and urban populations show clear separation.

Previous reports have indicated that Western populations have lower microbial richness than non-Western populations^3^. Our analyses of microbial richness yielded similar results. We used targeted amplification and sequencing of the V4 region of the 16S rRNA gene (Methods), followed by clustering of sequences into Operational Taxonomic Units (OTUs). We find that the Matses and Tunapuco populations have higher richness than the Norman population. The trend is observed with both phylogenetic (Faith’s phylogenetic diversity (PD)) and non-phylogenetic (observed species) richness metrics (Fig. 1a). Further, these differences in richness between traditional and industrialized societies are robust to OTU assignment strategy (Methods) and rarefaction, being detected with as few as 5,000 reads per sample (Supplementary Fig. 2). No significant differences in richness are observed between the two traditional populations. The magnitude of difference observed between phylogenetic and non-phylogenetic richness indices indicates that the gut microbiomes of traditional societies are composed of larger number of phylogenetically diverse taxa, while the gut microbiomes of industrialized societies are composed of fewer closely related taxa (Fig. 1a).

Next, we compared microbial community structure (beta diversity) among the three populations using Principal Coordinates Analysis (PCoA) transformation of weighted UniFrac^6^ distances (Fig. 1b). The traditional and industrialized populations show separation in PCoA space, and among the traditional populations the Matses form a separate cluster (PERMANOVA, *P*<0.001 and *P*<0.001 respectively). Further, the Tunapuco population is characterized by high interpersonal variation, evident in both PC axes 1 and 2. Supervised learning using Random Forests^7^, a machine learning method utilizing microbial community signatures, accurately assigned samples to their source population based on taxonomic profiles at the OTU level (100% accuracy, all populations).

### Taxonomic characterization

**Figure 2:**
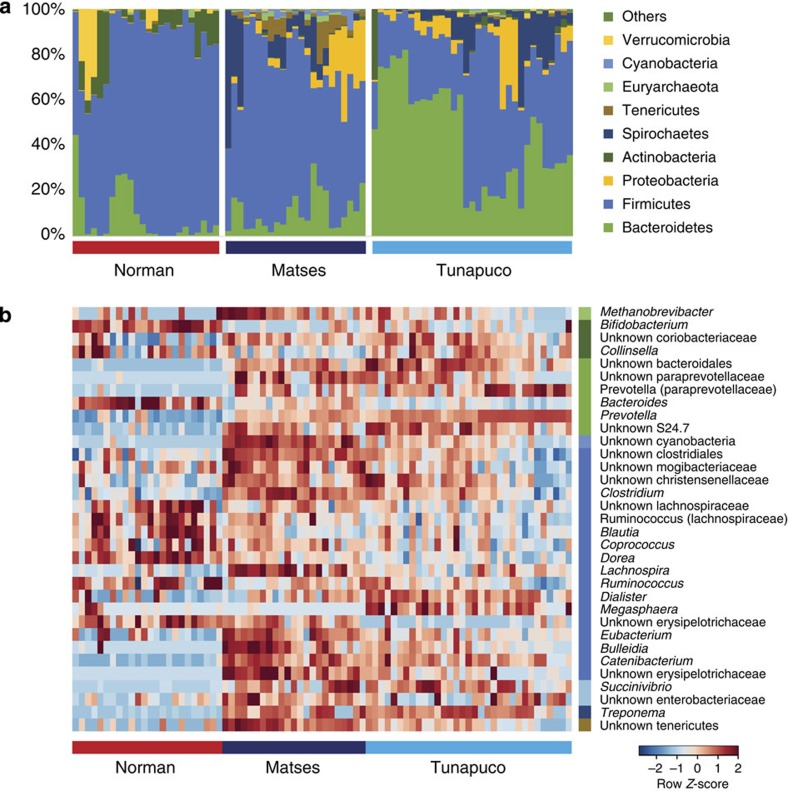
Taxonomic profile of the gut microbiomes of the Matses, Tunapuco and Norman populations. Analyses were performed on 16S rRNA V4 region data, rarefied to a depth of 10,000 reads per sample. (**a**) Relative taxa abundance plots for individuals from the three populations, summarized at the phylum level. Individuals are represented along the horizontal axis, and relative taxa frequency is denoted by the vertical axis. (**b**) Heatmap showing 33 genera with significant differences in abundance between populations (Kruskal–Wallis, FDR-corrected *P*<0.05). Individual boxplots for phyla and genera are shown in Supplementary Fig. 3. Heatmap is colour-coded based on row *z*-scores.

To test whether subsistence traditions harbour distinct microbial communities, we compared relative abundance of taxa between each of our populations. The three populations show differences in taxonomic distribution at the phylum level (Fig. 2a), with 8 out of 20 phyla having a significant difference in abundance in at least 1 population (False Discovery Rate (FDR)-corrected Kruskal–Wallis test: *P*<0.0006) (Supplementary Table 4). Three of the eight phyla show a traditional/urban-industrial distribution, with the traditional populations (Matses and Tunapuco) enriched for Proteobacteria and Spirochaetes and the urban-industrial population (Norman) enriched for Actinobacteria (Supplementary Fig. 3). In addition, the Matses differ from the Tunapuco and Norman populations in being enriched for Cyanobacteria, Tenericutes and Euryarchaeota (Supplementary Fig. 3). Finally, the Tunapuco population is enriched for Bacteroidetes, while the Norman and Matses populations are enriched for Firmicutes (Supplementary Fig. 3).

To further characterize taxonomic differences, we performed Kruskal–Wallis tests on genus-level taxa and identified 33 genera showing significant differences in abundance between the three populations (FDR-corrected Kruskal–Wallis test: *P*<0.05; Fig. 2b, Supplementary Table 5). The traditional/urban-industrial trends observed among Actinobacteria, Proteobacteria and Spirochaetes are driven by the genera *Bifidobacterium*, *Succinivibrio* and *Treponema*, respectively (Supplementary Fig. 3). While a high relative abundance of Bacteroidetes distinguishes Tunapuco from the Matses and Norman populations, at the genus level this is further resolved into a traditional/urban-industrial trend driven by higher levels of *Prevotella* among traditional gut microbiomes and *Bacteroides* among urban-industrial gut microbiomes. This pattern is similar to previous reports for non-Western populations^1^,^3^ (Supplementary Fig. 3). With respect to Firmicutes, we observe a complex pattern driven by the enrichment of different genera among the three populations. Specifically, the Norman population is enriched for *Ruminococcus*, *Blautia*, *Dorea* and an unknown genus in the family Lachnospiraceae (Supplementary Fig. 3). The Matses are enriched for *Clostridium*, *Catenibacterium*, *Eubacterium*, *Lachnospira* and an unknown genus in the class Clostridiales (Supplementary Fig. 3). The Tunapuco population, while overall having lower levels of Firmicutes, is specifically enriched for the genus *Dialister* (Supplementary Fig. 3). Overall, these taxa distribution patterns are concordant with trends reported from previous studies on hunter-gatherer and rural agriculturalist communities^1^,^3^.

To evaluate population discrimination, we performed supervised clustering using Random Forests on taxa tables summarized at higher taxonomic levels (genus to phylum). The Norman population consistently had a 100% classification accuracy at all taxonomic levels. In contrast, the Matses and Tunapuco populations had a 93% and 100% classification accuracy, respectively, at the genus level, reducing to 77% and 91% at the phylum level (Supplementary Table 6). Misclassification occurred exclusively between the rural populations, with samples being cross-assigned between the Matses and Tunapuco, indicating shared community signatures at higher taxonomic levels between these two populations.

**Figure 3:**
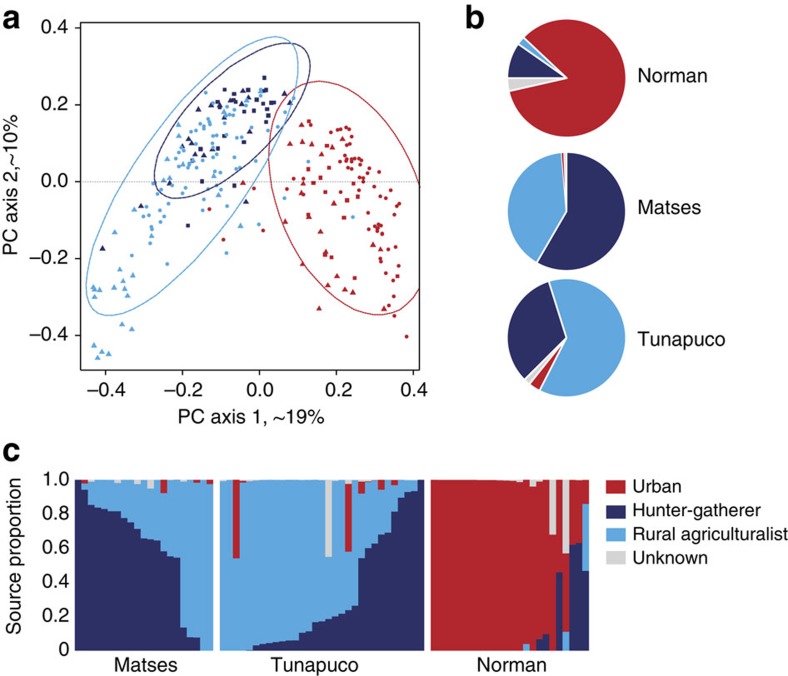
Comparison of the gut microbiomes of Matses, Tunapuco and Norman populations to published data from hunter-gatherer, rural agriculturalist and urban-industrial communities. Analyses were performed on genus-level taxa tables rarefied to 4,000 reads per sample. (**a**) Principal coordinate analysis of Bray–Curtis distances generated from taxa tables summarized at the genus level. Proportion of variance explained by each principal coordinate axis is denoted in the corresponding axis label. Populations are colour coded by subsistence strategy. Data sets are represented by triangles (this study), circles (Yatsunenko *et al.*^3^), and squares (Schnorr *et al.*^1^). Ellipses correspond to 95% confidence boundaries for each of the three subsistence categories. (**b**) Results from Bayesian source-tracking analysis. Source contributions are averaged across samples within the population. (**c**) Results from Bayesian source tracking for individual samples.

Finally, we compared genus-level taxa abundance profiles between our populations, and those from two previous studies of remote agrarian and hunter-gatherer human gut microbiomes^1^,^3^. PCoA of a Bray–Curtis distance matrix generated from genus-level taxa tables shows a clear separation between traditional and urban-industrial microbiomes (Fig. 3a), consistent across the three different studies. In addition, the hunter-gatherer populations (Matses and Hadza) form a distinct sub-cluster nested within the other traditional populations (Tunapuco, Venezuela and Malawi). To further explore this trend, we performed Bayesian source tracking^8^ on the Matses, Tunapuco and Norman samples using the previously published data sets as source populations (traditional hunter-gatherer: Hadza; rural agriculturalist: Venezuela, Malawi; and urban-industrial: USA, Italy; Fig. 3b). Consistent with previous analyses, the urban sources formed the primary contribution (~84% average) to the Norman samples, while the combined rural and hunter-gatherer sources accounted for ~95–98% for the Tunapuco and Matses samples. Specifically, the Matses samples had a higher contribution (~58%) from the Hadza hunter-gatherer source, while the Tunapuco samples had a higher contribution (~66%) from the rural Venezuela and Malawi source. Within populations, individuals show variation (Fig. 3c), but overall between ~64 and 85% of individuals have profiles consistent with their subsistence strategy. Thus, while the studies were conducted with differences in sample handling (freezing and desiccation), extraction methods (MoBio PowerSoil and phenol-chloroform) and choice of PCR primers, they nevertheless show a pattern in which two hunter-gatherer populations from two separate continents (Africa and South America) have a greater affinity to each other than to other traditional or urban populations. This is similarly true for the rural agriculturalists in Africa and South America and the urban industrial populations in Europe and North America.

### Functional characterization

We performed shotgun metagenome sequencing (Illumina, see Methods) to investigate whether the Matses, Tunapuco and Norman gut microbiomes harbour differences in functional capacity. To improve annotation quality, the short reads obtained from metagenome sequencing were assembled *de novo* using Ray-Meta^9^ to generate longer contigs (Methods). Functional capacity was then inferred from annotation of Open Reading Frames (ORFs) predicted from these contigs. We used an annotation pipeline incorporating microbial genomes (draft and complete) obtained from the HMP DACC^10^, IMG (v3.5; ref. 11), and NCBI GenBank databases^12^ as references.

**Figure 4:**
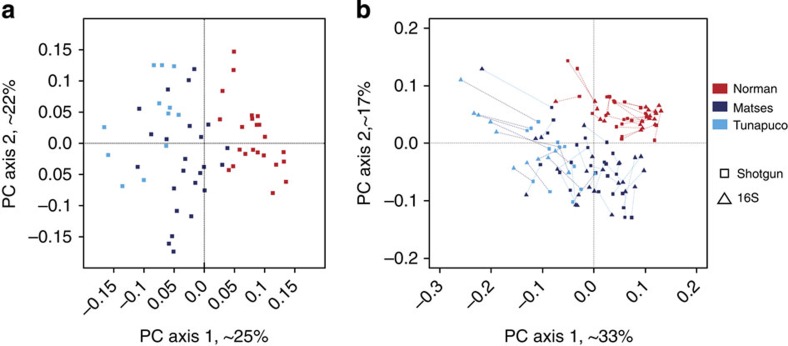
Comparison of taxonomic and functional diversity of gut microbiomes between populations. Proportion of variance explained by each principal coordinate axis is denoted in the corresponding axis label (**a**) Principal Coordinates Analysis of Bray–Curtis distances generated from KEGG Orthologue tables rarefied to 200,000 counts per sample. (**b**) Procrustes analysis between the taxonomic and the functional data sets on paired samples from the Matses, Tunapuco and Norman populations.

Supervised clustering using KEGG Orthology (KO)^13^,^14^ profiles distinguished the traditional and urban-industrial populations with 100% accuracy (Supplementary Table 7). Within the traditional populations, the Matses samples had a 100% classification accuracy, while 1 Tunapuco sample (out of 12) was misassigned to the Matses. Beta-diversity plots generated from Bray–Curtis distance matrices (PC transformed) of KO tables showed a clear separation between the traditional and urban-industrial populations (Fig. 4a). Procrustes analyses comparing spatial fit between PC plots generated from UniFrac (taxonomic) and Bray–Curtis (functional) distances showed concordance, indicating consistency between taxonomic and functional profiles (Fig. 4b).

To identify KOs showing differential abundance between the three populations, we performed Kruskal–Wallis tests on KO tables. Overall, we identified 112 KOs showing a significant difference in abundance in at least 1 population (Supplementary Table 8). Of these, 78 KOs (69.6%) show enrichment among the traditional populations; these KOs are predominantly associated with metabolism and genetic information processing. Among the remaining KOs, 20 (17.8%) show enrichment specific to the urban-industrial population and 14 (12.5%) show similar distributions between the urban-industrial and at least one of the two traditional populations. The KOs uniquely enriched in the urban-industrial populations are predominantly associated with membrane transport functions. In addition, 37 of the 78 KOs enriched in the traditional populations are found at higher abundance among the residents of Tunapuco compared with the Matses.

**Figure 5:**
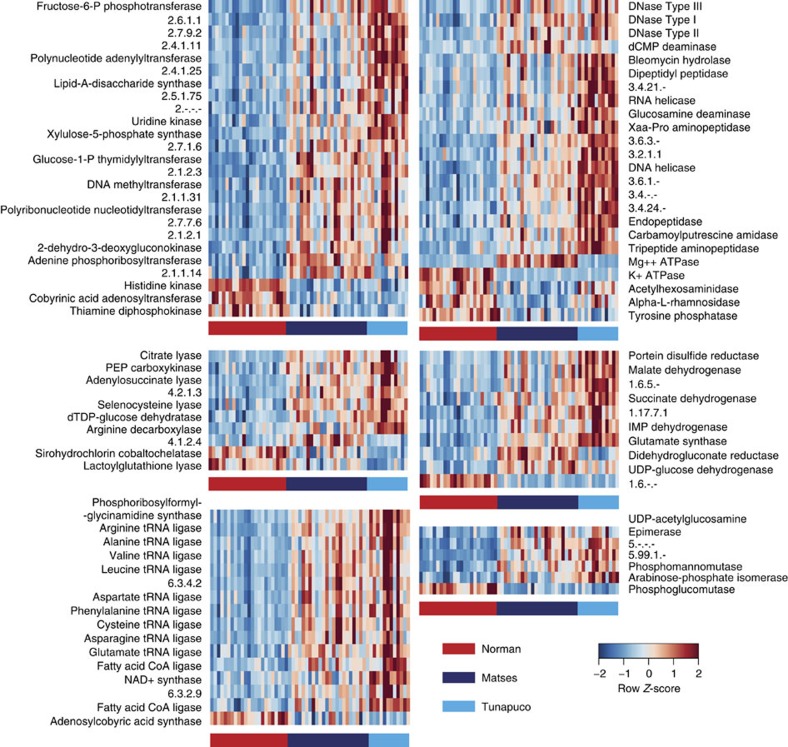
Heatmap of ECs showing significant differences between the gut microbiomes of Matses, Tunapuco and Norman populations. Enzymes are grouped based on EC class. Comparisons between populations were performed using Kruskal–Wallis tests (FDR-corrected *P*<0.05). Heatmap is colour coded based on row *z*-scores.

To further characterize some of these functional differences, we performed statistical analyses on orthologue tables annotated using Enzyme Commission (EC) codes^15^. Overall, we identified 91 ECs showing significant differences between the populations (Fig. 5, Supplementary Table 9). Of these, 79 ECs (86.8%) are enriched among the traditional populations, including several associated with the Tricarboxylic acid cycle (for example, succinate dehydrogenase and malate dehydrogenase) and amino acid metabolism (for example, amino acid transfer RNA ligases). These pathways are related to enhanced capacity for energy production and dietary amino acid uptake. Similar to our observations with the KOs, a subset of 39 ECs show higher abundance within Tunapuco compared with the Matses. Further, a second group comprised of 34 ECs is enriched within a subset of individuals from both the Matses and Tunapuco. The remaining 12 ECs (13.2%) were enriched in the Norman population and included 3 ECs related to Vitamin B1 and B12 biosynthesis.

### Age stratification and *Bifidobacterium*

A previous study on US, Malawi and Venezuelan populations^3^ found that age resulted in a significant gradient of bacterial abundances, with newborns initially showing high variation but little differentiation among populations, and eventually resembling the adults of their respective communities by 3 years of age^3^,^16^. Further, this trend was shown to correlate with the abundance of *Bifidobacterium*, a genus thought to be associated with dietary dairy consumption. As the number of children of age <3 years in our study is limited to four individuals from the Matses, we instead performed correlation analyses between age and PC axes generated from a weighted UniFrac distance matrix. A negative correlation was observed between the first PC axis and age for the Matses population (*ρ*=−0.59, *P*<0.002). While the relative abundance of *Bifidobacterium* in children shows no direct correlation with age, 10 out of 13 individuals (total *n*=25) showing presence of the genus were <7 years old. In contrast, all individuals sampled from our Norman population showed presence of *Bifidobacterium*, with no correlation between age and levels of *Bifidobacterium*. This is consistent with regular dairy consumption self-reported by the Norman individuals.

### *Treponema* and rural populations

Although Spirochaetes have been previously reported from the gut microbiome of non-human primates^17^,^18^,^19^ and ancient human populations^20^, they have only been observed in high abundance among extant human populations with non-Western lifestyles, such as a traditional community in Burkina Faso^1^ and a hunter-gatherer community in Tanzania^1^. As such, they may represent a part of the human ancestral gut microbiome that has been lost through the adoption of industrial agriculture and/or other lifestyle changes (Supplementary Table 10). Similar to previous studies on traditional populations, we find that both the Matses and Tunapuco are enriched for Spirochaetes, specifically of the genus *Treponema*. Phylogenetic analysis of these Spirochaetes indicates the presence of at least five *Treponema* OTUs (Supplementary Table 11, Supplementary Fig. 4) found in traditional populations today. Of these, two OTUs (Greengenes 13.5 OTU ids: 300310, 338950) occur at high frequencies and are shared between the Matses and Tunapuco, and a third OTU (Greengenes 13.5 OTU id: 4307383) is present at high frequencies in the Tunapuco population but is rare among the Matses. The phylogenetic similarity of these OTUs with *Treponema succinifaciens*, a non-pathogenic carbohydrate metabolizer and a member of the swine gut microbiome^21^, offers support to the hypothesis that these organisms might be selected for under high fibre diets.

**Figure 6:**
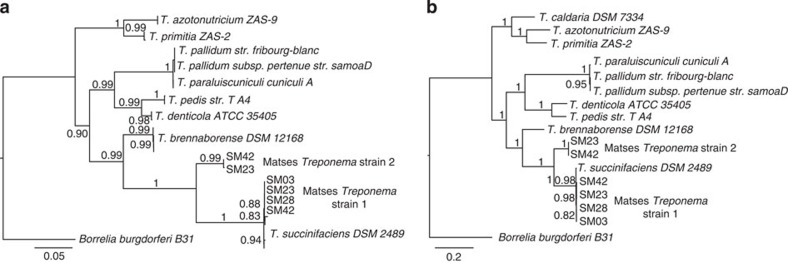
Phylogenetic trees showing relationship of Matses *Treponema* strains to reference *Treponema* species. (**a**) Maximum likelihood tree constructed using 16S rRNA sequences from *de novo* assemblies of shotgun data. (**b**) Maximum likelihood tree constructed using concatenated amino acid sequences from 35 single copy marker loci, retrieved from *de novo* assemblies of shotgun data. Both trees show similar topology, with the Matses *Treponema* strains grouping with *Treponema succinifaciens*, a known carbohydrate metabolizer in the swine gut microbiome.

**Figure 7:**
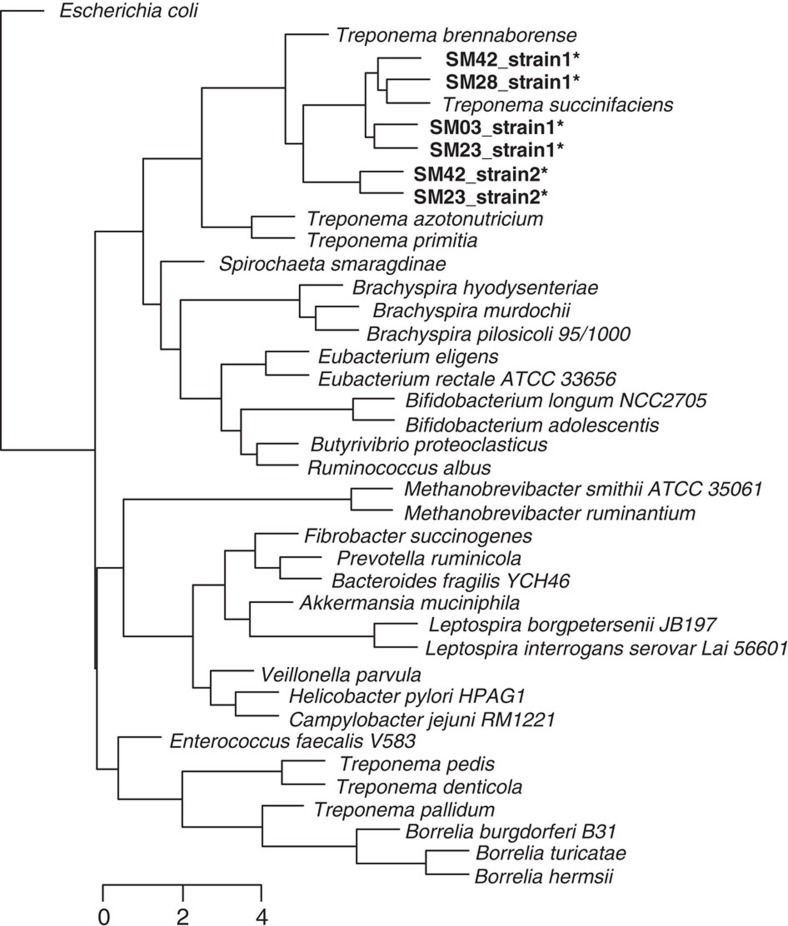
Hierarchical clustering of Matses *Treponema* and reference bacterial strains based on KEGG functional potential data. Open reading frames (ORFs) predicted from reconstructed Matses *Treponema* genomes were annotated using the MAPLE server^24^ and compared with reference bacterial genomes (including Spirochaetes). The Matses *Treponema* strains share functional similarities with *Treponema succinifaciens*, a known carbohydrate metabolizer and apathogenic member of the swine gut microbiome. *denotes the Matses *Treponema* strains.

To further characterize the phylogenetic and functional relationships of the Matses gut *Treponema* to other currently available reference strains from this genus, we retrieved contigs matching *Treponema* from metagenomes assembled *de novo* (Methods) from four Matses samples. These samples were selected based on high frequencies of *Treponema* observed in their taxonomic profiles. Samples from Tunapuco were not included in this analysis as they had lower sequencing coverage and often contained multiple *Treponema* strains leading to poor assemblies. Phylogenetic analysis using 16S rRNA gene sequences retrieved from these contigs confirmed the presence of two distinct strains of *Treponema* within these samples, one with ~99% sequence similarity to *T. succinifaciens* (found in all four samples, referred to as Strain 1) and the other with ~90% sequence similarity to *T. succinifaciens* (found in two samples, referred to as Strain 2) (Fig. 6a). A second phylogenetic tree constructed using concatenated amino acid sequences from 35 single copy marker loci^22^ (predominantly composed of ribosomal small and large subunit proteins) showed similar topology, confirming the presence of two distinct strains of *Treponema* within our samples (Fig. 6b). Overall, we retrieved between 2.19 and 2.46 Mb of genome sequence data for the *Treponema* strains through a combination of methods, including sequence identity to the reference *T. succinifaciens*, GC% and coverage statistics (Methods). We annotated these partial assemblies using the ‘prokka’ pipeline^23^, followed by evaluation of metabolic potential using MAPLE^24^. We then performed hierarchical clustering using metabolic Module Completion Ratio (MCR) data obtained from the MAPLE^24^ pipeline (Fig. 7). Based on predicted metabolic potential, the reconstructed *Treponema* strains cluster most closely with *T. succinifaciens* and are nested with other gut-associated treponemes reported from termites (*T. azotonutricium* and *T. primitia*)^25^ and a digital dermatitis associated *Treponema* reported from cattle (*T. brennaborense*)^26^. In addition, these strains functionally cluster with gut-associated members of the *Brachyspira* clade of Spirochaetes, along with several gut-associated bacteria from other phyla, including *Ruminococcus*, *Eubacterium* and *Butyrivibrio*. In contrast several pathogenic Spirochaetes including *T. pallidum* (syphilis), *Borrellia burgdorferi* (Lyme disease) and *T. denticola* (periodontal disease), form a functionally separate clade outside of the gut-associated bacteria. Overall, these results give further support for a potential metabolic role for the *Treponema* strains observed in the gut microbiomes of traditional human populations.

## Discussion

Characterizing microbial communities and their functions in populations living relatively ancestral lifestyles is essential for understanding the coevolution of humans as a species with their microbiomes. Our results strongly support the need for human microbiome research on a larger sampling of human lifeways and traditions. Such work with vulnerable populations is challenging, especially with respect to building trust and establishing reasonable informed consent, but remains possible, even with very remote and traditional peoples. Without these insights, the benefit of research may be more applicable to the Westernized, affluent, urban populations, further exacerbating health disparities for the underrepresented. Here we present a microbiome profile that may be more consistent with the ancestral state of human biology. Such information provides a potential foundation for understanding microbiome-associated ‘diseases of civilization’.

## Methods

### Community engagement

Collaborative research with remote human communities requires careful planning and extensive outreach. As with many other indigenous populations, the Matses and Tunapuco have experienced and resent the idea of safari or helicopter research, a common model of research on indigenous populations. In addition, foreign companies’ recent attempts to extract oil from the Matses’ natural reservation have fuelled the Matses’ distrust towards the outside world. To maximize protection of the communities, we consulted with colleagues at the Center for Intercultural Health of the Peruvian Institute of Health from the early stages of the study design.

Recognizing communities’ vulnerabilities and concerns, in addition to the official efforts aiming to protect them, we initiated our work by engaging political and traditional authorities in the review of our protocol. Political authorities included regional and national authorities. The traditional authority we first approached was the Peruvian leader of the ethnic group. All concerns from these authorities were addressed in the protocol before submission to the ethics committee of the Peruvian National Institute of Health, which approved the protocol.

The protocol for the Matses community includes oversight by additional local authorities. On protocol approval, and with the authorization of the leader of the Matses ethnic group, we presented our project to the local authority of the District Yaquerana, who oversees all activities in the Matses reservation, and later to the leader of the Comunidad Nativa Matses Anexo San Mateo, who introduced us to the community members. Such structures are unavailable for Tunapuco. For both communities, we implemented a public meeting for community consultation and obtained community consent. In addition to community consent, all volunteer participants were individually consented when they arrived to deposit their samples.

In an effort to maximize benefits and prevent potentially coercive incentives for the community, we avoided individual presents or compensation. Instead, we offered on-site parasite screening, making a microscope available for the community to observe the analysis we performed. This experience also served to anchor the discussion about microorganisms, emphasizing the informed part of the consent process. A Matses interpreter, who was fluent in Spanish, mediated communication with the Matses community.

For both the Matses and Tunapuco communities, once preliminary results became available we returned to the community to disseminate our findings. We obtained authorization from the community for data publication and to use the community’s name in association with our findings.

### Sample collection and processing

Faecal samples from participants were collected in polypropylene containers. Samples from the Matses (*n*=25) and Tunapuco populations (*n*=31) were stored in ice for up to 4 days until arriving at Lima, and they were kept frozen until DNA was extracted at the laboratory in Oklahoma. In addition, faecal samples were collected from 23 individuals from Norman, Oklahoma to serve as a comparative population with an urban-industrial lifestyle. These samples were kept on ice during transport to the laboratory and frozen within 24 h of collection.

DNA extraction from the Matses and Tunapuco faecal samples was performed using the PowerSoil DNA Isolation Kit (MoBio) following manufacturer’s instructions, with the addition of two heating steps: 10 min at 60 °C before vortexing the samples with the PowerBeads and later 10 min at 90 °C. For the Norman faecal samples, DNA extraction was performed using the PowerMicrobiome RNA Isolation Kit (MoBio) with the exclusion of the DNase I step. Both extraction methods included an initial bead-beating step.

To characterize the taxonomic profile of the gut microbiome, we amplified the V4 hypervariable region of the bacterial 16S rRNA gene using the universal bacterial/archaeal primers F515 (5′-CACGGTCGKCGGCGCCATT-3′) and R806 (5′-GGACTACHVGGGTWTCTAAT-3′)^27^. These same primers were used to generate 16S rRNA data in a previous study of agrarian and urban gut microbiomes^3^. A 12 bp GOLAY error-correcting barcode was added to the reverse primer to enable sample multiplexing. Reactions were performed in triplicate using the AccuPrime Taq DNA Polymerase High Fidelity system. Read statistics from the 16S V4 sequencing runs are summarized in Supplementary Table 3. To characterize gut microbiome functional potential, we performed shotgun metagenomic sequencing of faecal samples. Libraries were prepared using the Nextera DNA sample preparation kit for NGS libraries (Illumina platform).

### 16S sequencing data processing

The 16S rRNA sequencing data from the Illumina runs were filtered and trimmed using the programme ‘sickle’ ( https://github.com/najoshi/sickle) to remove bases with a quality score <30, followed by discarding sequences with ambiguous bases (‘N’) and a length <90 bp. These trimmed reads were demultiplexed, chimera filtered (‘usearch’)^28^, and assigned to OTUs using packages implemented in QIIME^29^. We initially performed closed-reference OTU assignment using ‘uclust’^28^ with a 97% sequence similarity threshold against the Greengenes 13.5 database^30^ as a reference. Overall, >95% of the total sequences were assigned to OTUs using this approach, with the urban population from Norman having ~97±2% and rural Matses and Tunapuco populations having ~96±2% and ~95±3%, respectively, assigned to OTUs. In addition, to document the impact of potentially novel OTUs on microbial richness, the remaining unassigned sequences were clustered *de novo* using a 97% sequence similarity threshold, and the resulting OTU table merged with the one generated using the closed-reference approach. Comparative 16S rRNA data sets were obtained from previously published studies^1^,^3^, and are composed of hunter-gatherers (Hadza, *n*=27), rural agriculturalists (Venezuela, *n*=60; Malawi, *n*=20) and urban populations (USA, *n*=65; Italy, *n*=16). The data set composed of Venezuela, Malawi and USA individuals^3^ had been sequenced on an Illumina platform and were processed using the same quality filtering and OTU assignment criteria as employed by this study. The data set composed of the Hadza and Italian individuals^1^ had been sequenced on a Roche 454 platform, and were processed using QIIME’s *de novo* clustering strategy using a 97% sequence similarity threshold to maximize read assignment to OTUs. All comparisons between sequences generated in this study and the two previously published data sets are limited to genus-level taxa tables.

### Shotgun read processing

The data sets generated from shotgun metagenome sequencing were quality filtered and trimmed to remove bases with a quality score <30, followed by discarding sequences with ambiguous bases (‘N’) and a length <25 bp. *De novo* metagenome assembly was performed on these trimmed sequences using Ray Meta^9^, with a k-mer length of 21. Metagenome assembly was performed on the OU Supercomputing Center for Education and Research (OSCER) platform. ORF prediction was performed on assembled contigs using ‘FragGeneScan’^31^. Predicted ORFs were assigned annotations through comparisons with 382 gut microbial genomes from the Human Microbiome Project (HMP DACC)^10^,^32^. Unmapped ORFs were then compared sequentially to JGI’s Integrated Microbial Genomes data set^11^(IMG, v 3.50, 12 October 2012), followed by sequenced microbial genomes from NCBI^12^. Annotations were performed using the ‘ublast’ module implemented in ‘usearch’^28^, with a sequence identity threshold of 60%, query coverage fraction of 50% and e-value of 1e–5. Assembly and annotation statistics are summarized in Supplementary Table 12. Depth of coverage for contigs was calculated through mapping of raw reads to assembled contigs using Bowtie2 (ref. 33), followed by processing using ‘samtools’^34^ and custom R scripts. Median depth of coverage over the entire contig was then assigned as its abundance. Biological Observation Matrix (BIOM) files^35^ were created incorporating ORF abundance, and annotation using the KO^13^ information and EC^15^ codes. These BIOM files were subsequently used for comparisons of functional potential between the three populations.

### Data analyses

Alpha diversity analyses were performed using observed species and PD indices, as implemented in QIIME^29^. Beta-diversity analyses were performed using weighted UniFrac^6^ (16S rRNA), and Bray–Curtis (Genus tables, shotgun KO) distance metrics, as implemented in QIIME. Statistical analyses including PCoA, PERMANOVA tests, supervised machine learning (Random Forest)^7^,^36^ and Bayesian source-tracking^8^ were performed in QIIME^29^. Comparison of taxonomic and functional counts data between the three populations were performed using Kruskal–Wallis tests with multiple testing correction (FDR) implemented in R. Boxplots, heatmaps and two-dimensional PCoA plots were generated using R^37^. PERMANOVA were performed using 1,000 permutations to estimate *P* values for differences among categories (that is, country). Machine learning analyses utilized Random Forest classifiers with 10-fold cross-validation and 1,000 trees.

### *Treponema* genome reconstruction

Contigs assembled from shotgun metagenomic reads obtained from four Matses individuals (SM03, SM23, SM28 and SM42) were screened for 16S rRNA gene sequences and 35 single copy marker loci sequences^22^ using a combination of NCBI-BLAST^38^,^39^ and Hidden Markov Models (HMM)^40^ profile searches. Contigs with best matches within the *Treponema* genus were filtered. Two strains of *Treponema* were identified in our samples. Strain 1, found in all four samples, had a >99% sequence identity to *T. succinifaciens* at the 16S rRNA locus (nucleotide) and 35 single copy marker loci (average, amino acid). The second strain (Strain 2), found in samples SM23 and SM42 had ~90–91% sequence identity (nucleotide) at the 16S rRNA locus and ~88% sequence identity (average, amino acid) at the single copy marker loci to *T. succinifaciens*. Several of the single copy marker loci co-assembled on contigs. Depth of coverage was consistent for marker loci on different contigs. Further, in samples with both strains (SM23 and SM42) the strains were observed to have different depths of coverage, consistently observed across their respective contigs. Additional contigs were assigned to the two strains using a combination of NCBI-BLAST, depth of coverage and GC%. Assembly evaluation was performed using the ‘reapr’ pipeline^41^. Assembly statistics are presented in Supplementary Table 13. Functional analysis and annotation were performed on filtered contigs using the ‘prokka’ pipeline^23^. Predicted ORFs were submitted through the MAPLE server^24^, to evaluate functional potential. The functional potential (MCR, KEGG pathways) of the Matses *Treponema* strains were compared using hierarchical clustering with a collection of reference genomes, including other Spirochaetes and several gut-associated bacteria across other phyla.

## Additional information

**Accession codes:** 16S rRNA gene sequences from the study have been deposited in the QIIME database under study id 1448 (Illumina HiSeq V4). Shotgun sequence data sets have been deposited in the NCBI SRA database under the BioProject id PRJNA268964.

**How to cite this article:** Obregon-Tito, A. J. *et al.* Subsistence strategies in traditional societies distinguish gut microbiomes. *Nat. Commun.* 6:6505 doi: 10.1038/ncomms7505 (2015).

## Supplementary Material

Supplementary Information Supplementary Figures 1-4, Supplementary Tables 1-13 and Supplementary References
