# Supplementary material for: Subsistence strategies in traditional societies distinguish gut microbiomes
Source: Nat Commun. 2015 Mar 25;6:6505. doi: 10.1038/ncomms7505 (PMC4386023; doi:10.1038/ncomms7505)
Supplement: Supplementary Information — Supplementary Figures 1-4, Supplementary Tables 1-13 and Supplementary References [file ncomms7505-s1.pdf]

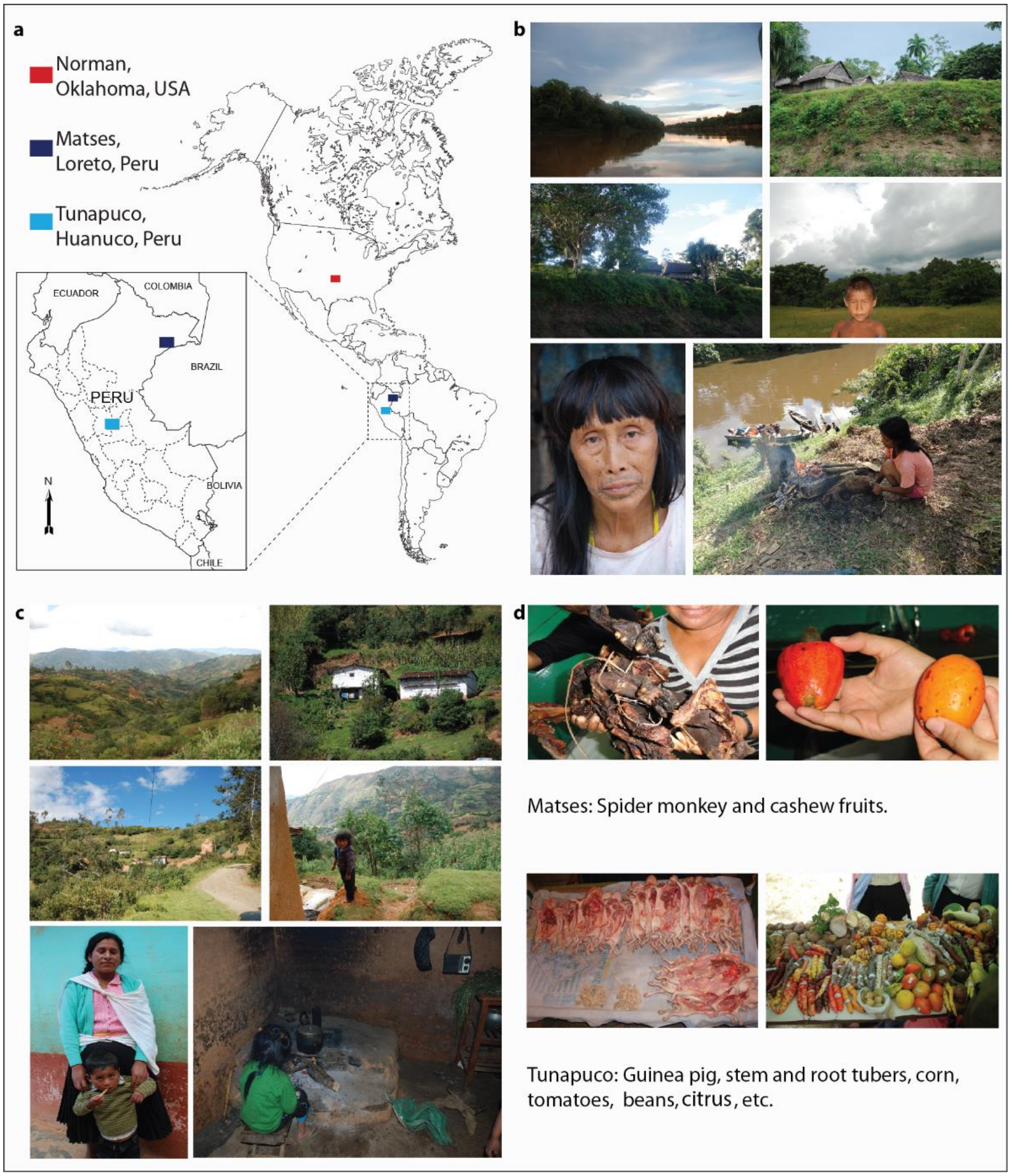

Supplementary Figure 1| Geography and lifestyle of the Matses and Tunapuco traditional Peruvian human populations. (a) Map showing the populations represented in this study. (b) The Matses community of San Mateo is composed of 14 hunter-gatherer families settled close together along the Peruvian Amazon River at 150 meters above the sea level (masl). (c) Tunapuco is an agricultural community composed of numerous families spread over a large area in the Central Andes region, with one to two family units occupying each hill at a variety of elevations ranging from 2500-3100 masl. (d) The Matses and Tunapuco diets are based on local resources, but have distinct faunal and floral composition. Our control Western population is a community of university-associated residents living in Norman, Oklahoma (357 masl).

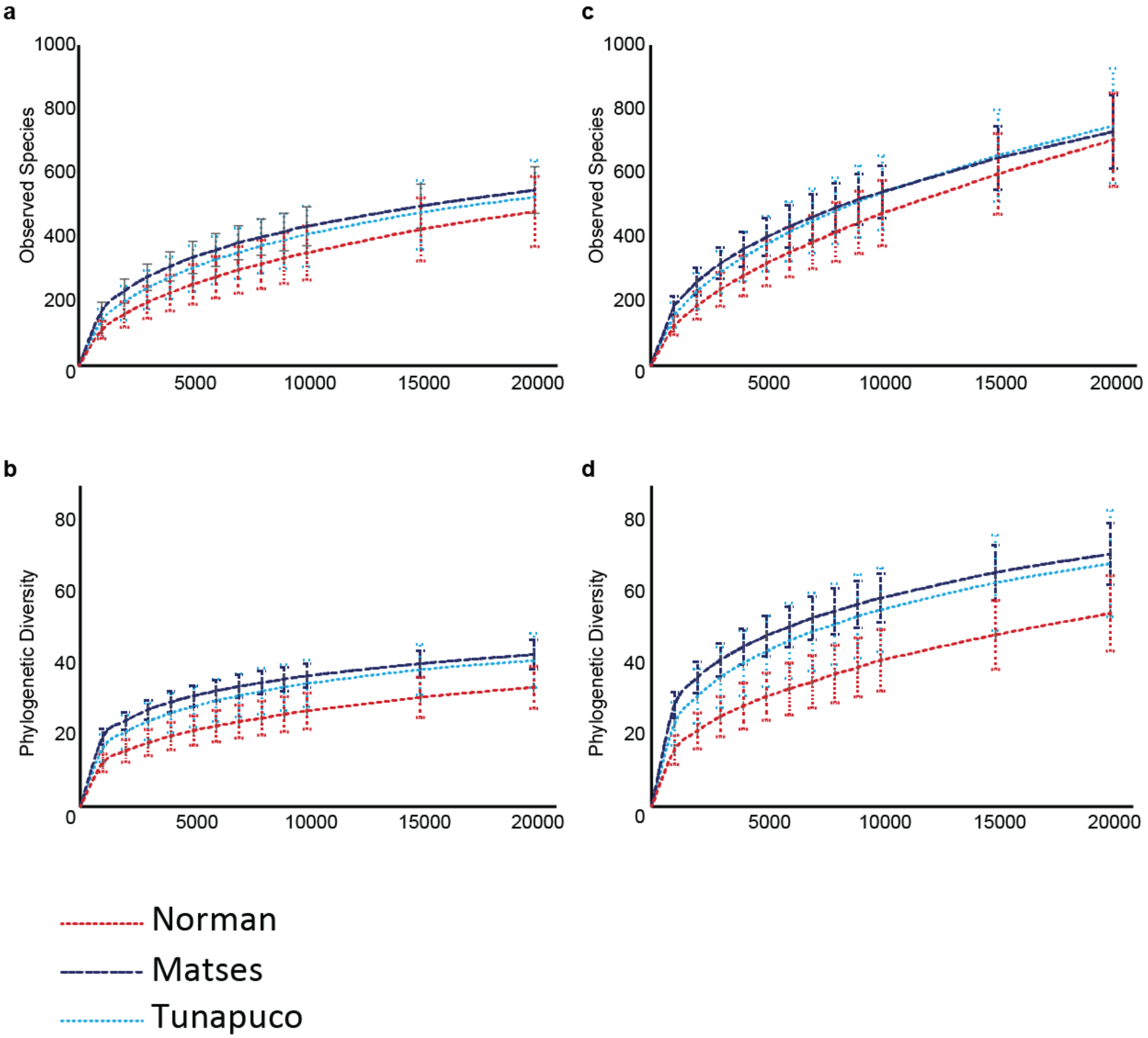

Supplementary Figure 2| Rarefaction curves for gut microbiome richness estimates in the three study populations. Rarefaction curves were generated using both closed reference (a, b) and open reference (c, d) OTU tables, and were calculated with non-phylogenetic (a, c) and phylogenetic (b, d) metrics. Note that the differences among the three populations are greatest when analyzed with respect to phylogenetic diversity. Horizontal and vertical axes represent rarefaction depth and alpha diversity values respectively. Error bars correspond to standard deviation for alpha diversity values within a population at each rarefaction depth.

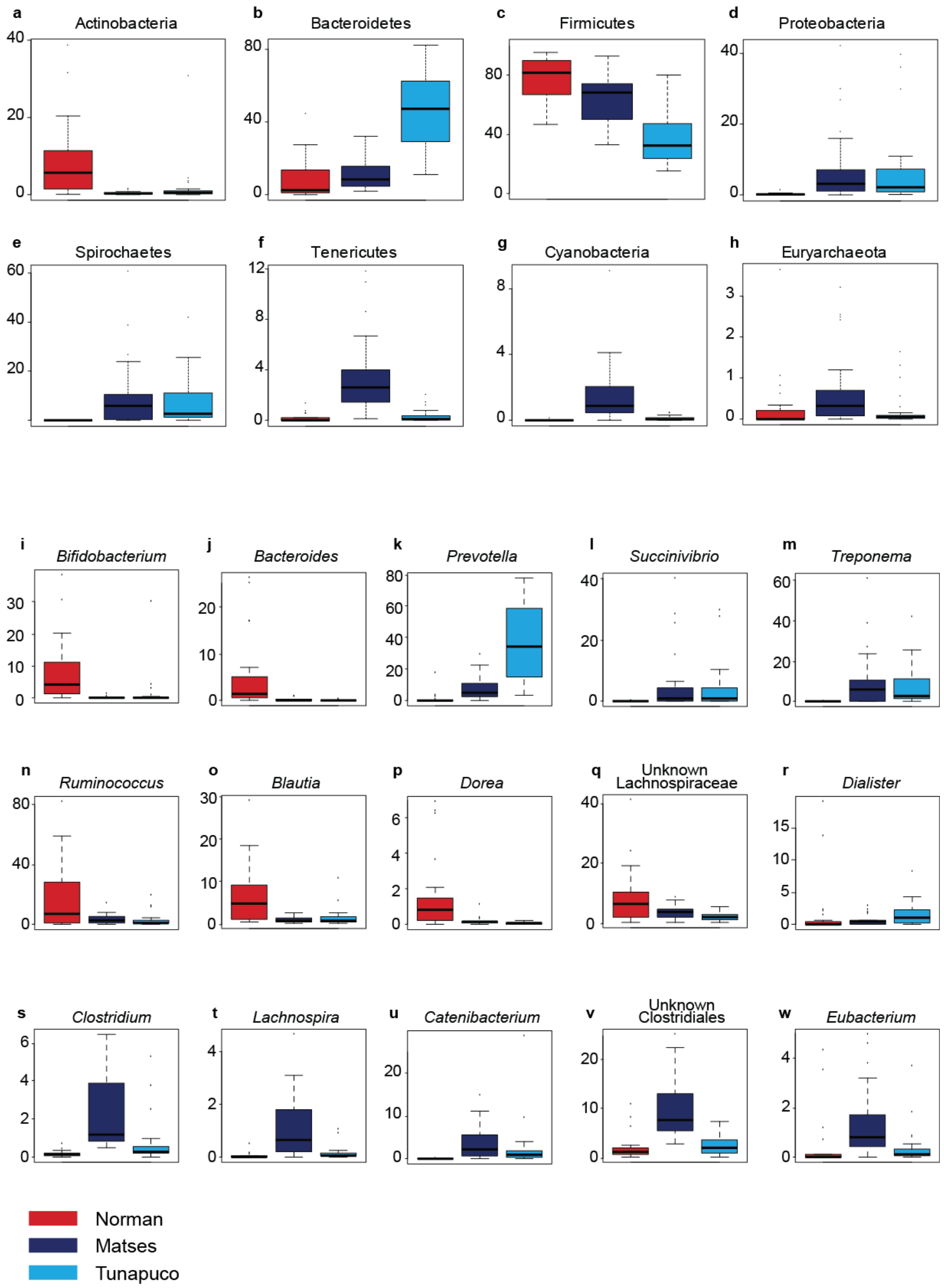

Supplementary Figure 3| Boxplots for taxa exhibiting significantly different (Kruskal-Wallis, FDR-adjusted *P* < 0.05) relative abundances among the three study populations with respect to phylum (a-h) and genus (i-w). The vertical axis represents relative abundance in percentage. Whiskers in the boxplot represent the range of minimum and maximum values within a population, excluding outliers.

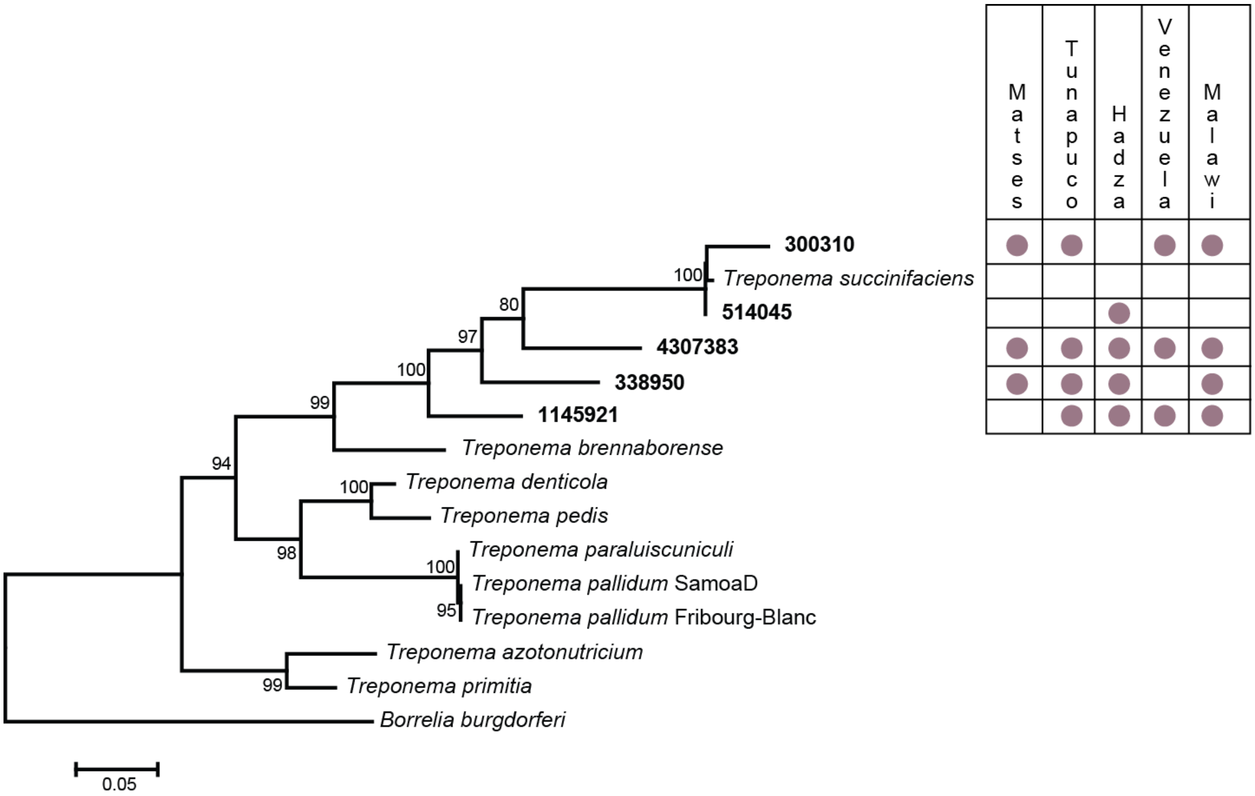

Supplementary Figure 4| Maximum likelihood tree illustrating the phylogenetic position of human gut-microbiome associated *Treponema* OTUs. Among Spirochaetes, human-associated gut treponemes form a distinct clade that also includes *T. succinifaciens*, a swine-associated gut treponeme, but which is distinct from insect-associated gut treponemes (*T. azotonutricium, T. primitia*), dermatitis-associated treponemes in cattle (*T. brennaborense, T. pedis*), human oral treponemes (*T. denticola*), and other pathogenic Spirochaetes (*T. pallidum, B. burgdorferi*). To date, a total of 5 human gut treponeme OTUs have been identified, and their occurrence (presence/absence) in diverse human populations is indicated by shaded circles. Note that OTUs 300310 and 514045 may represent the same organism, which is very closely related to *T. succinifaciens*. Bootstrap values above 80% (generated from 1,000 replicates) are indicated on the tree.

Supplementary Table 1. Summary of the Matses diet. Nutritional data was collected using a food frequency questionnaire. Respondents generally stated that their diet is dependent upon availability. Plantain and manioc, which are rudimentarily cultivated in small plots around their settlement, are the only constants in the Matses diet, with six or more portions consumed each day. Similar to other Amazonian groups, fish appears to be their main source of protein, supplemented by other wild game, such as monkey, and sloth.

						Nutritional content (per 100 g)		
	**Matses name**	**Common name**	**Scientific name**	**Frequency**	**Mode of consumption**	**Protein**	**Fat**	**Carbohydrates**
**Staple food, main source of calories**	Pachid ushu	Varieties of manioc	*Manihot* spp.	Everyday / anytime	Boiled	0.48	0.1	31
	Pachid piu				Drink (Masato)	0.2	0.1	8.9
	Pachid taui chëshë				Fermented and toasted (farina)	No data available. It is estimated that 45% of protein and sucrose is lost during fermentation. Starch digestibility is increased by fermentation.		
	Mani	Varieties of plantain/banana	*Musa* spp.		Raw (average of varieties)	1.2	0.27	25
	Mani bënë							
	Manimbo							
	Mani chëshë				Chapo (main drink, accompanies all meals)	Approximately a third of the nutritional content of raw banana (volumetric dilution)		
	Mani ushu							
	Mani chotac							
	Mani masquë				Boiled	0.7	0.3	36.8
	Mani macho							
	Mani piu				grilled	1.5	0.2	68
	Mani tadan							

Supplementary Table 1 (cont.) Summary of the Matses diet

	Matses name	Common name	Scientific name	Frequency	Mode of consumption	Nutritional content (per 100 g)		
						Protein	Fat	Carbohydrates
Other crops	Cadi piu	Sweet potato	*Ipomoea batata*	Couple times a month	Boiled, grilled	1.6	0.2	26.1
	Cadi chëshë							
	Piacbo	Maize	*Zea mays*	Monthly	Boiled, grilled	3.3	0.8	27.8
	Piacbo bëdi							
	Chotacquëna bëaid	Guaba, orange, mangoes. Lemon, grapefruit, rice, cacao, etc. Any crop that is brought from outside the community	various	Sometimes, when available	Various	NA	NA	NA
Mammals	Poshto	Woolly monkey	*Lagothrix lagotricha*	Few times a month	Grilled, smoked, boiled	NA	NA	NA
	Achu	Howler monkey	*Alouatta seniculus*			NA	NA	NA
	Shuinte	Sloth	*Bradypus sp*			NA	NA	NA
	Shëshëid	Spider monkey	*Ateles sp*			NA	NA	NA
	Shëcten	Collared peccary	*Tayassu tajacu*			85.6	NA	NA
	Shëctenamë	White-lipped peccary	*Tayassu pecari*			84.6	NA	NA

Supplementary Table 1 (cont.) Summary of the Matses diet

	Matses name	Common name	Scientific name	Frequency	Mode of consumption	Nutritional content (per 100 g)		
						Protein	Fat	Carbohydrates
Reptiles	Piush	Yellow-footed tortoise	*Geochelone denticulata*	Few times a month	Grilled, smoked, boiled	84.4	NA	NA
	Zeta	Yellow-spotted river turtle	*Podocnemis unifilis*			NA	NA	NA
Birds	Cushu	Trinidad piping guan	*Aburria pipile*	Few times a month	Grilled, smoked, boiled	NA	NA	NA
	Mando	Grey-winged trumpeter	*Psophia sp*			NA	NA	NA
	Uesnid	Razor-billed Curassow	*Mitu tuberosa*			NA	NA	NA
Fish	Sabalo (*)	Sabalo	*Prochilodus lineatus*	Daily/Few times a week	boiled, grilled or cooked inside leaves (Patarashca)	NA	NA	NA
	Maparate (*)	Maparate	*Hypophthalmus marginatus*			15.1	0.9	NA
	Lisa (*)	Leaping mullet	*Liza saliens*			20.8	3.3	0
	Tucunare (*)	Tucanare peacock bass	*Cichla monocullus*			NA	NA	NA
	Boquichico (*)	Black prochilodus	*Prochilodus nigricans*			47.9	9.1	0
Eggs		Turtle, alligator		Irregular.	(Average values)	15.1	11.3	2.9

Supplementary Table 2. Summary of the Tunapuco diet. Nutritional data was collected using a food frequency questionnaire.

	Common name	English description	Scientific name	Frequency	Mode of consumption	Nutritional content (per 100 g) Protein	Fat	Carbohydrates
Staple food, main source of calories	Papa	Potato	*Solanum tuberosa*	Everyday / anytime	Boiled	2.1	0.1	22.3
	Chuno, Tunta	Dehydrated potatos			Boiled	1.9	0.5	77.7
	Oca	Andean tuber	*Oxalis tuberosa*		Boiled	1	0.6	13.3
	Pan andino	Artisan Bread	-		Oven cooked	9.6	0.3	71.8
	Mote	Hominy	*Zea mays*		Boiled	5.9	2.1	78.3
	Arroz	Rice	*Oryza sativa*	Daily/a few times a week	Boiled	7.8	0.7	77.6
	Tocosh de papa	Fermented potatoes	*Solanum tuberosa*	Daily	Boiled	NA		
	Ajos	Garlic	*Allium sativum*	Few times a week	Fried/boiled	5.6	0.8	30.4
	Zanahoria	Carrots	*Daucus carota*		Boiled	2.4	0.3	8.1
	Col	Cabagges	*Brassica oleracea*		Boiled/Stewed	1.5	0.3	4.9
	Habas	Faba beans	*Vicia faba*		Boiled	11.3	0.8	25.9
Fruits	Manzana	Apple	*Malus domestica*	A few of these a week (Seasonal)	Raw	0.3	0.1	14.6
	Naranja	Orange	*Citrus sinensis*		Raw	0.6	0.2	10.1
	Pepino	Melon pear	*Solanum muricatum*		Raw	0.3	0	7
	Mandarina	Tangerine	*Citrus reticulata*		Raw	0.6	0.3	8.6
	Platano	banana	*Musa paradisiaca*		Raw	1.5	0.3	21
	Mango	Mango	*Mangifera indica*		Raw	0.4	0.2	15.9
	Piña	Pineapple	*Ananas comosus*		Raw	0.4	0.2	9.8
	Papaya	Papaya	*Carica papaya*		Raw	0.4	0.1	8.2

Supplementary Table 2 (cont.) Summary of the Tunapuco diet

	Common name	English description	Scientific name	Frequency	Mode of consumption	Nutritional content (per 100 g)		
						Protei n	Fat	Carbohydrates
Mammals	Cuy	Guinea Pig	*Cavia porcellus*	Biweekly	Boiled/Stewed/Fried	19	1.6	0.1
	Vaca	Beef	*Bos taurus*	Monthly	Boiled/Stewed	21.3	1.6	0
	Chancho	Pork	*Sus scrofa domesticus*	Biweekly	Boiled/Stewed/Fried	14.4	15.1	0.1
	Carnero	Sheep	*Ovis aries*	Couple times a week	Boiled/Stewed/Fried	16.9	21.6	0
Birds	Pollo/gallina	Chicken/hen	*Gallus gallus domesticus*	Weekly	Boiled/Stewed/Fried	21.4	3.1	0
	Huevos de gallina	Eggs		Variable, biweekly	Boiled	12.9	8.4	1.9
Fish	Jurel	Horse mackerel	*Trachurus murphyi*	Seasonal	Fried	19.7	4	0.1
	Trucha	Rainbow trout	*Oncorhynchus mykiss*	Monthly	Stewed/Fried	19.5	3.1	0
Diary	Leche fresca de vaca	Natural fresh milk		Weekly	Raw/Boiled	3.1	3.5	4.8
	Queso Fresco de vaca	Fresh white cheese		Weekly	Raw/added to dishes	15.8	17.5	2.2

Supplementary Table 2 (cont.) Summary of the Tunapuco diet

	Common name	English description	Scientific name	Frequency	Mode of consumption	Nutritional content (per 100 g)		
						Protein	Fat	Carbohydrates
Store bought products	Avena	Oatmeal	*Avena Sativa*	Weekly	Boiled	1.3	0.5	10.9
	Fideos	Pasta		Twice a week	Boiled	9.4	0.2	78.2
	Azucar	Sugar	*Industrial sucrose*	Weekly	Added to oatmeal, tea, cofee at breakfast	0	0	99.1
	Pimienta	Black Pepper	*Piper nigrum*	Sparsely used	Seasoning	Minute quantities, No contribution to energy		
	Comino	Cumin	*Cuminum cyminum*		Seasoning			
	Leche evaporada	Canned condensed milk		Weekly	Diluted with hot water	7	8.1	10.9
	Aceite	Cooking oil		Very small quantities a few times a week	To fry garlic or some proteins	0	100	0

Supplementary Table 3. Participant metadata. De-identified information includes sample IDs, age, sex, body mass index (BMI), BMI class, and types of analyses performed. BMI was calculated according to the formula BMI=W/H^2, where 'W' is weight in Kilograms, and 'H' is height in meters. Sample IDs listed here are concordant with the labels used in 16S rRNA and shotgun datasets.

<table>
<tr><th rowspan="2">Sample</th><th rowspan="2">Population</th><th rowspan="2">Age</th><th rowspan="2">Sex</th><th rowspan="2">BMI</th><th rowspan="2">BMI Class</th><th rowspan="2">16S rRNA</th><th rowspan="2">Shotgun Metagenome</th><th rowspan="2">Read Depth 16S</th><th colspan="2">Read Depth Shotgun</th></tr>
<tr><th>Paired</th><th>Single</th></tr>
<tr><td>**HCO01**</td><td>**Tunapuco**</td><td>36</td><td>Female</td><td>17.85</td><td>Underweight</td><td>Yes</td><td>No</td><td>25,890</td><td colspan="2">NA</td></tr>
<tr><td>**HCO02**</td><td>**Tunapuco**</td><td>9</td><td>Female</td><td>14.05</td><td>Healthy</td><td>Yes</td><td>Yes</td><td>29,142</td><td>3,046,571</td><td>342,289</td></tr>
<tr><td>**HCO03**</td><td>**Tunapuco**</td><td>6</td><td>Male</td><td>15.68</td><td>Healthy</td><td>Yes</td><td>No</td><td>19,922</td><td colspan="2" rowspan="2">NA</td></tr>
<tr><td>**HCO04**</td><td>**Tunapuco**</td><td>4</td><td>Male</td><td>16.16</td><td>Healthy</td><td>Yes</td><td>No</td><td>26,615</td></tr>
<tr><td>**HCO07**</td><td>**Tunapuco**</td><td>3</td><td>Female</td><td>19.67</td><td>Obese</td><td>Yes</td><td>Yes</td><td>28,915</td><td>5,411,239</td><td>817,165</td></tr>
<tr><td>**HCO09**</td><td>**Tunapuco**</td><td>13</td><td>NA</td><td>18.77</td><td>Healthy</td><td>Yes</td><td>Yes</td><td>32,715</td><td>5,212,204</td><td>477,186</td></tr>
<tr><td>**HCO10**</td><td>**Tunapuco**</td><td>10</td><td>Male</td><td>19.43</td><td>Overweight</td><td>Yes</td><td>No</td><td>28,935</td><td colspan="2"></td></tr>
<tr><td>**HCO11**</td><td>**Tunapuco**</td><td>36</td><td>Male</td><td>23.05</td><td>Healthy</td><td>Yes</td><td>Yes</td><td>106,266</td><td>4,990,441</td><td>644,918</td></tr>
<tr><td>**HCO12**</td><td>**Tunapuco**</td><td>35</td><td>Female</td><td>27.01</td><td>Overweight</td><td>Yes</td><td>Yes</td><td>19,869</td><td>4,674,029</td><td>677,003</td></tr>
<tr><td>**HCO13**</td><td>**Tunapuco**</td><td>9</td><td>Female</td><td>18.12</td><td>Healthy</td><td>Yes</td><td>No</td><td>175,400</td><td colspan="2" rowspan="8">NA</td></tr>
<tr><td>**HCO14**</td><td>**Tunapuco**</td><td>34</td><td>Female</td><td>31.97</td><td>Obese</td><td>Yes</td><td>No</td><td>86,378</td></tr>
<tr><td>**HCO15**</td><td>**Tunapuco**</td><td>63</td><td>Female</td><td>25.11</td><td>Healthy</td><td>Yes</td><td>No</td><td>170,387</td></tr>
<tr><td>**HCO16**</td><td>**Tunapuco**</td><td>11</td><td>NA</td><td>17.70</td><td>Healthy</td><td>Yes</td><td>No</td><td>180,819</td></tr>
<tr><td>**HCO17**</td><td>**Tunapuco**</td><td>7</td><td>Female</td><td>14.65</td><td>Healthy</td><td>Yes</td><td>No</td><td>179,419</td></tr>
<tr><td>**HCO18**</td><td>**Tunapuco**</td><td>11</td><td>Male</td><td>19.68</td><td>Healthy</td><td>Yes</td><td>No</td><td>220,801</td></tr>
<tr><td>**HCO21**</td><td>**Tunapuco**</td><td>10</td><td>Male</td><td>17.47</td><td>Healthy</td><td>Yes</td><td>No</td><td>165,947</td></tr>
<tr><td>**HCO41**</td><td>**Tunapuco**</td><td>54</td><td>NA</td><td>25.45</td><td>Overweight</td><td>Yes</td><td>No</td><td>263,049</td></tr>
<tr><td>**HCO53**</td><td>**Tunapuco**</td><td>44</td><td>Female</td><td>29.52</td><td>Overweight</td><td>Yes</td><td>Yes</td><td>19,010</td><td>3,400,837</td><td>2,001,102</td></tr>
<tr><td>**HCO61**</td><td>**Tunapuco**</td><td>20</td><td>Female</td><td>NA</td><td>NA</td><td>Yes</td><td>Yes</td><td>115,066</td><td>6,056,934</td><td>867,376</td></tr>
<tr><td>**HCO62**</td><td>**Tunapuco**</td><td>NA</td><td>Male</td><td>50.51</td><td>Obese</td><td>Yes</td><td>No</td><td>160,478</td><td colspan="2" rowspan="2">NA</td></tr>
<tr><td>**HCO63**</td><td>**Tunapuco**</td><td>6</td><td>Female</td><td>24.37</td><td>Obese</td><td>Yes</td><td>No</td><td>249,229</td></tr>
<tr><td>**HCO64**</td><td>**Tunapuco**</td><td>NA</td><td>Female</td><td>NA</td><td>NA</td><td>Yes</td><td>Yes</td><td>31,265</td><td>6,653,729</td><td>735,161</td></tr>
</table>

Supplementary Table 3 (cont.) Participant metadata.

Sample	Population	Age	Sex	BMI	BMI Class	16s rRNA	Shotgun Metagenome	Read Depth 16S	Read Depth Shotgun	
									Paired	Single
HCO65	Tunapuco	NA	Female	NA	NA	Yes	No	25,429	NA	
HCO66	Tunapuco	11	Male	17.33	Healthy	Yes	No	37,801		
HCO67	Tunapuco	26	Female	19.53	Healthy	Yes	Yes	33,269	5,597,578	840,511
HCO68	Tunapuco	7	Male	16.57	Healthy	Yes	No	211,072	NA	
HCO69	Tunapuco	9	NA	18.55	Healthy	Yes	No	98,768		
HCO70	Tunapuco	40	Female	NA	NA	Yes	Yes	134,329	6,698,447	761,803
HCO72	Tunapuco	5	Female	20.36	Obese	Yes	Yes	209,579	3,513,166	3,183,823
HCO73	Tunapuco	NA	Female	NA	NA	Yes	No	177,601	NA	
HCO74	Tunapuco	36	Female	31.53	Obese	Yes	Yes	144,051	6,025,093	563,713
SM01	Matses	30	Male	NA	NA	Yes	Yes	96,773	35,415,317	4,290,766
SM02	Matses	25	Female	23.95	Healthy	Yes	Yes	89,370	45,077,030	4,730,237
SM03	Matses	10	Male	15.77	Healthy	Yes	Yes	108,481	22,223,170	2,499,433
SM05	Matses	1	Male	NA	NA	Yes	Yes	97,170	23,064,470	2,912,751
SM10	Matses	6	Female	17.82	Healthy	Yes	No	86,656	NA	
SM11	Matses	4	Female	15.78	Healthy	Yes	Yes	99,468	32,172,764	3,851,086
SM18	Matses	36	Female	26.44	Overweight	Yes	Yes	103,438	31,120,212	3,476,246
SM20	Matses	20	Female	21.27	Healthy	Yes	Yes	95,462	29,059,286	3,486,160
SM23	Matses	7	Male	15.66	Healthy	Yes	Yes	99,439	29,246,497	3,356,893
SM24	Matses	2	Male	17.85	Healthy	Yes	Yes	99,989	32,150,556	3,362,732
SM25	Matses	2	Female	NA	NA	Yes	Yes	282,526	30,670,037	2,781,980
SM28	Matses	52	Female	19.47	Healthy	Yes	Yes	93,951	26,916,930	2,296,893
SM29	Matses	50	Female	17.9	Underweight	Yes	Yes	105,303	29,679,776	2,503,068
SM30	Matses	4	Male	18.26	Healthy	Yes	Yes	90,000	27,403,057	2,492,589

Supplementary Table 3 (cont.) Participant metadata.

Sample	Population	Age	Sex	BMI	BMI Class	16S rRNA	Shotgun Metagenome	Read Depth 16S	Read Depth Shotgun	
									Paired	Single
**SM31**	**Matses**	30	Male	22.6	Healthy	Yes	Yes	111,057	29,763,287	2,808,360
**SM32**	**Matses**	21	Female	22.14	Healthy	Yes	Yes	91,905	34,345,773	3,796,280
**SM33**	**Matses**	5	Female	16.45	Healthy	Yes	Yes	93,080	28,011,827	3,085,715
**SM34**	**Matses**	4	Male	15.62	Healthy	Yes	Yes	111,553	26,523,695	2,805,036
**SM37**	**Matses**	12	Male	19.84	Healthy	Yes	Yes	109,898	26,897,725	3,208,320
**SM39**	**Matses**	40	Female	28.93	Overweight	Yes	Yes	52,181	33,171,349	3,592,545
**SM40**	**Matses**	18	Female	22.72	Healthy	Yes	Yes	83,169	22,027,536	4,731,158
**SM41**	**Matses**	6	Male	NA	NA	Yes	Yes	85,973	30,018,788	4,505,169
**SM42**	**Matses**	4	Male	16.73	Healthy	Yes	Yes	109,175	27,856,765	3,847,805
**SM43**	**Matses**	2	Female	15.15	Healthy	Yes	Yes	85,863	29,093,620	7,985,469
**SM44**	**Matses**	4	Male	19.83	Healthy	Yes	Yes	107,827	29,109,143	7,918,170
**NO01**	**Norman**	23	Male	21.69	Healthy	Yes	Yes	219,925	20,625,472	1,981,126
**NO02**	**Norman**	37	Female	20.52	Healthy	Yes	Yes	123,772	25,977,359	2,216,831
**NO03**	**Norman**	40	Male	23.37	Healthy	Yes	Yes	84,042	26,854,797	2,338,379
**NO04**	**Norman**	26	Male	24.16	Healthy	Yes	Yes	120,143	23,801,292	2,203,115
**NO05**	**Norman**	28	Male	22.19	Healthy	Yes	Yes	125,909	24,207,515	2,162,375
**NO06**	**Norman**	28	Male	23.49	Healthy	Yes	Yes	148,940	19,877,728	1,709,795
**NO07**	**Norman**	32	Female	21.92	Healthy	Yes	No	357,828	NA	
**NO08**	**Norman**	32	Female	20.01	Healthy	Yes	Yes	234,531	21,375,588	1,542,028
**NO09**	**Norman**	34	Female	23.77	Healthy	Yes	Yes	137,595	18,797,354	1,438,450
**NO10**	**Norman**	41	Male	26.58	Overweight	Yes	Yes	171,714	23,486,818	1,605,024
**NO11**	**Norman**	26	Male	23.93	Healthy	Yes	Yes	163,147	25,017,951	1,757,924
**NO12**	**Norman**	27	Female	28.62	Overweight	Yes	Yes	139,647	24,281,027	1,701,998
**NO13**	**Norman**	35	Male	20.34	Healthy	Yes	Yes	73,907	22,261,146	1,590,912

Supplementary Table 3 (cont.) Participant metadata.

Sample	Population	Age	Sex	BMI	BMI Class	16S rRNA	Shotgun Metagenome	Read Depth 16S	Read Depth Shotgun	
									Paired	Single
**NO14**	**Norman**	10	Female	14.97	Healthy	Yes	Yes	201,279	23,161,751	1,800,520
**NO15**	**Norman**	50	Female	25.92	Overweight	Yes	Yes	137,201	20,524,182	1,801,257
**NO16**	**Norman**	47	Male	30.86	Obese	Yes	Yes	207,056	28,327,953	2,251,370
**NO17**	**Norman**	10	Male	21.53	Overweight	Yes	Yes	152,005	21,377,881	1,692,516
**NO18**	**Norman**	7	Male	17.31	Healthy	Yes	Yes	133,136	24,510,902	1,920,710
**NO19**	**Norman**	32	Female	19.30	Healthy	Yes	Yes	169,580	23,047,857	1,888,095
**NO20**	**Norman**	26	Male	27.86	Overweight	Yes	Yes	144,511	22,716,185	1,786,034
**NO21**	**Norman**	23	Male	24.78	Healthy	Yes	Yes	136,702	22,567,813	1,840,925
**NO22**	**Norman**	26	Male	30.22	Obese	Yes	Yes	133,508	24,490,144	1,997,954
**NO23**	**Norman**	26	Female	26.53	Overweight	Yes	Yes	157,919	26,588,425	2,307,414

Supplementary Table 4. Summary of phyla with significantly different relative abundances (Kruskal-Wallis, FDR-adjusted *P* < 0.05) among the three study populations.

Phylum	p-value	FDR adjusted p-value	Median frequency (%) within populations		
			Norman	Matses	Tunapuco
Euryarchaeota	0.0003	0.0006	0.0	0.3	0.1
Actinobacteria	0	0	5.7	0.3	0.5
Bacteroidetes	0	0	2.6	8.3	47.2
Cyanobacteria	0	0	0.0	0.9	0.1
Firmicutes	0	0	81.5	68.0	32.3
Proteobacteria	0	0	0.2	3.2	2.2
Spirochaetes	0	0	0.0	5.8	2.8
Tenericutes	0	0	0.0	3.0	0.2

Supplementary Table 5. Summary of genera with significantly different relative abundances (Kruskal-Wallis, FDR-adjusted *P* < 0.05) among the three study populations.

Taxonomy		*P*-value	FDR adjusted *P*-value	Median frequency (%) within populations		
Phylum	Genus			Norman	Matses	Tunapuco
Euryarchaeota	*Methanobrevibacter*	0.0001	0.0005	0.0	0.3	0.0
Actinobacteria	*Bifidobacterium*	0	0	3.7	0.0	0.0
Actinobacteria	Unknown Coriobacteriaceae	0.0011	0.0034	0.0	0.2	0.2
Actinobacteria	*Collinsella*	0.0008	0.0026	0.2	0.0	0.1
Bacteroidetes	Unknown Bacteroidales	0	0	0.0	0.2	0.4
Bacteroidetes	Unknown Paraprevotellaceae	0	0	0.0	0.1	0.0
Bacteroidetes	*Prevotella** (Paraprevotellaceae)	0	0	0.0	0.1	0.3
Bacteroidetes	*Bacteroides*	0	0	1.4	0.1	0.0
Bacteroidetes	*Prevotella*	0	0	0.0	5.5	32.7
Bacteroidetes	Unknown Bacteroidales	0	0	0.0	0.5	1.2
Cyanobacteria	Unknown YS2	0	0	0.0	0.9	0.1
Firmicutes	Unknown Clostridiales	0	0	1.3	7.8	2.2
Firmicutes	Unknown Mogibacteriaceae	0.0003	0.001	0.0	0.1	0.0
Firmicutes	Unknown Christensenellaceae	0	0.0002	0.1	0.6	0.2
Firmicutes	*Clostridium*	0	0	0.1	1.2	0.3
Firmicutes	Unknown Lachnospiraceae	0.0007	0.0023	7.3	4.1	2.1
Firmicutes	*Ruminococcus** (Lachnospiraceae)	0	0.0001	1.3	0.4	0.2
Firmicutes	*Blautia*	0.0019	0.0055	3.9	0.9	0.9
Firmicutes	*Coprococcus*	0.0154	0.037	0.8	0.7	0.4
Firmicutes	*Dorea*	0	0	0.9	0.1	0.1
Firmicutes	*Lachnospira*	0	0	0.0	0.9	0.1
Firmicutes	*Ruminococcus*	0.0052	0.0138	7.4	2.0	1.4
Firmicutes	*Dialister*	0.0002	0.0009	0.0	0.5	1.1
Firmicutes	*Megasphaera*	0	0	0.0	0.0	0.1
Firmicutes	Unknown Erysipelotrichaceae	0	0	0.3	0.2	0.0
Firmicutes	*Eubacterium*	0	0	0.0	0.8	0.1
Firmicutes	*Bulleidia*	0	0	0.0	0.2	0.0
Firmicutes	*Catenibacterium*	0	0	0.0	2.3	0.9
Firmicutes	Unknown Erysipelotrichaceae (P75.A5)	0	0	0.0	0.2	0.0
Proteobacteria	*Succinivibrio*	0	0	0.0	0.8	0.8
Proteobacteria	Unknown Enterobacteriaceae	0.0006	0.0021	0.0	0.4	0.1
Spirochaetes	*Treponema*	0	0	0.0	5.8	2.8
Tenericutes	Unknown Mollicutes	0	0	0.0	2.5	0.2

Supplementary Table 6. Accuracy of supervised classification at different phylogenetic levels expressed as percentages.

Taxonomic Level	Norman	Matses	Tunapuco
Phylum (L2)	100	73	91
Class (L3)	100	87	94
Order (L4)	100	87	94
Family (L5)	100	91	97
Genus (L6)	100	91	100
OTU (L7)	100	100	100

Supplementary Table 7. Confusion Matrix displaying accuracy of supervised classification based on KEGG Orthology (KO).

		Predicted			
		Norman	Matses	Tunapuco	Classification accuracy (%)
Actual	Norman	23	0	0	100
	Matses	0	24	0	100
	Tunapuco	0	1	11	92

Supplementary Table 8. Summary of KEGG Orthology (KO) ids with significantly different relative abundances (Kruskal-Wallis, FDR-adjusted *P* < 0.05) among the three study populations.

KEGG ORTHOLOGY	*P*	FDR-corrected *P*	Norman	Matses	Tunapuco	Function
K00265	0.00	0.00	151.00	200.00	264.50	Metabolism; Amino Acid Metabolism; Alanine, aspartate and glutamate metabolism; K00265
K00266	0.00	0.00	195.00	273.00	314.50	Metabolism; Amino Acid Metabolism; Alanine, aspartate and glutamate metabolism; K00266
K00527	0.00	0.00	189.00	267.00	289.00	Metabolism; Nucleotide Metabolism; Purine/Pyrimidine metabolism; K00527
K00540	0.00	0.02	566.00	608.00	671.50	Unclassified; Metabolism; Others; K00540
K00548	0.00	0.01	187.00	172.50	225.50	Metabolism; Amino Acid Metabolism; Cysteine and methionine metabolism; K00548
K00558	0.00	0.01	266.00	370.50	290.50	Metabolism; Amino Acid Metabolism; Cysteine and methionine metabolism; K00558
K00599	0.00	0.00	649.00	687.50	541.50	Metabolism; Amino Acid Metabolism; Histidine/Tyrosine metabolism; K00599
K00615	0.00	0.01	220.00	284.50	246.50	Metabolism; Carbohydrate Metabolism; Pentose phosphate pathway; K00615
K00688	0.00	0.00	222.00	265.00	296.50	Metabolism; Carbohydrate Metabolism; Starch and sucrose metabolism; K00688
K00700	0.00	0.00	179.00	182.50	212.00	Metabolism; Carbohydrate Metabolism; Starch and sucrose metabolism; K00700
K00705	0.00	0.00	162.00	221.00	299.50	Metabolism; Carbohydrate Metabolism; Starch and sucrose metabolism; K00705
K00754	0.00	0.00	560.00	455.00	498.00	Metabolism; Carbohydrate Metabolism; Fructose and mannose metabolism; K00754
K00764	0.00	0.00	161.00	193.50	215.00	Metabolism; Amino Acid Metabolism; Alanine, aspartate and glutamate metabolism; K00764
K00936	0.00	0.00	1214.00	807.50	748.50	Unclassified; Metabolism; Others; K00936
K00951	0.00	0.00	171.00	204.50	263.50	Metabolism; Nucleotide Metabolism; Purine metabolism; K00951
K01006	0.00	0.00	129.00	200.50	173.50	Metabolism; Carbohydrate Metabolism; Pyruvate metabolism; K01006
K01043	0.00	0.00	84.00	121.00	213.50	Unclassified; Metabolism; Others; K01043
K01153	0.00	0.00	237.00	402.50	455.50	Unclassified; Genetic Information Processing; Restriction enzyme; K01153
K01154	0.00	0.00	147.00	213.50	240.00	Unclassified; Genetic Information Processing; Restriction enzyme; K01154
K01176	0.00	0.00	83.00	125.50	214.50	Metabolism; Carbohydrate Metabolism; Starch and sucrose metabolism; K01176
K01187	0.00	0.00	240.00	239.00	323.50	Metabolism; Carbohydrate Metabolism; Starch and sucrose metabolism; K01187
K01190	0.00	0.01	552.00	437.50	575.00	Metabolism; Glycan Biosynthesis and Metabolism; Other glycan degradation; K01190
K01338	0.00	0.00	162.00	194.50	237.50	Metabolism; Enzyme Families; Peptidases; K01338
K01362	0.01	0.03	261.00	222.00	227.00	Unclassified; Metabolism; Amino acid metabolism; K01362
K01417	0.00	0.00	207.00	258.50	272.50	Unclassified; Metabolism; Others; K01417
K01447	0.01	0.03	202.00	170.50	132.50	Unclassified; Cellular Processes and Signaling; Membrane and intracellular structural molecules; K01447
K01534	0.00	0.00	172.00	239.50	198.50	Unclassified; Metabolism; Energy metabolism; K01534
K01537	0.00	0.00	291.00	296.50	373.00	Unclassified; Metabolism; Others; K01537
K01552	0.00	0.00	248.00	326.50	319.00	Unclassified; Metabolism; Energy metabolism; K01552
K01662	0.00	0.00	138.00	180.00	237.50	Metabolism; Metabolism of Terpenoids and Polyketides; Terpenoid backbone biosynthesis; K01662
K01681	0.00	0.00	124.00	184.00	209.50	Metabolism; Carbohydrate Metabolism; Citrate cycle (TCA cycle); K01681

**Supplementary Table 8 (cont.) Summary of KEGG Orthology**						
**KEGG ORTHOLOGY**	***P***	**FDR-corrected *P***	**Norman**	**Matses**	**Tunapuco**	**Function**
K01710	0.00	0.00	137.00	187.00	211.50	Metabolism; Metabolism of Terpenoids and Polyketides; Polyketide sugar unit biosynthesis; K01710
K01740	0.00	0.00	187.00	237.00	230.00	Metabolism; Amino Acid Metabolism; Cysteine and methionine metabolism; K01740
K01784	0.00	0.00	185.00	255.00	252.50	Metabolism; Carbohydrate Metabolism; Amino sugar and nucleotide sugar metabolism; K01784
K01834	0.00	0.00	245.00	301.00	318.00	Metabolism; Carbohydrate Metabolism; Glycolysis / Gluconeogenesis; K01834
K01840	0.00	0.00	143.00	208.50	214.50	Metabolism; Carbohydrate Metabolism; Fructose and mannose metabolism; K01840
K01869	0.00	0.00	143.00	212.50	239.50	Metabolism; Amino Acid Metabolism; Valine, leucine and isoleucine biosynthesis; K01869
K01870	0.00	0.00	171.00	221.50	262.50	Metabolism; Amino Acid Metabolism; Valine, leucine and isoleucine biosynthesis; K01870
K01872	0.00	0.00	142.00	187.50	227.00	Genetic Information Processing; Translation; Aminoacyl-tRNA biosynthesis; K01872
K01873	0.00	0.00	156.00	217.50	217.00	Metabolism; Amino Acid Metabolism; Valine, leucine and isoleucine biosynthesis; K01873
K01874	0.00	0.00	134.00	174.50	210.00	Metabolism; Amino Acid Metabolism; Amino acid related enzymes; K01874
K01876	0.00	0.00	135.00	215.50	201.00	Genetic Information Processing; Translation; Aminoacyl-tRNA biosynthesis; K01876
K01887	0.00	0.00	114.00	161.50	205.50	Genetic Information Processing; Translation; Aminoacyl-tRNA biosynthesis; K01887
K01890	0.00	0.00	127.00	156.50	210.00	Genetic Information Processing; Translation; Aminoacyl-tRNA biosynthesis; K01890
K01897	0.00	0.00	107.00	151.00	242.00	Metabolism; Lipid Metabolism; Fatty acid metabolism; K01897
K01915	0.00	0.00	230.00	291.00	324.00	Metabolism; Amino Acid Metabolism; Alanine, aspartate and glutamate metabolism; K01915
K01952	0.00	0.00	168.00	222.50	292.50	Metabolism; Nucleotide Metabolism; Purine metabolism; K01952
K01955	0.00	0.00	236.00	313.00	400.50	Metabolism; Nucleotide Metabolism; Pyrimidine metabolism; K01955
K02004	0.00	0.00	843.00	610.00	651.50	Environmental Information Processing; Membrane Transport; Transporters; K02004
K02014	0.01	0.02	686.00	453.00	817.50	Unclassified; Cellular Processes and Signaling; Pores ion channels; K02014
K02025	0.00	0.00	349.00	324.50	202.50	Environmental Information Processing; Membrane Transport; Transporters; K02025
K02026	0.00	0.00	314.00	280.50	188.50	Environmental Information Processing; Membrane Transport; Transporters; K02026
K02027	0.00	0.00	212.00	126.00	122.00	Environmental Information Processing; Membrane Transport; Transporters; K02027
K02030	0.00	0.00	263.00	304.50	221.00	Environmental Information Processing; Membrane Transport; Transporters; K02030
K02335	0.00	0.00	134.00	168.00	257.00	Metabolism; Nucleotide Metabolism; Purine/Pyrimidine metabolism; K02335
K02337	0.00	0.00	172.00	250.00	310.00	Metabolism; Nucleotide Metabolism; Purine/Pyrimidine metabolism; K02337
K02355	0.00	0.00	324.00	402.50	458.50	Genetic Information Processing; Translation; Translation factors; K02355
K02469	0.00	0.00	247.00	319.50	351.50	Genetic Information Processing; Replication and Repair; DNA replication proteins; K02469
K02470	0.00	0.00	220.00	297.50	281.50	Genetic Information Processing; Replication and Repair; DNA replication proteins; K02470
K02483	0.00	0.00	266.00	202.00	119.00	Environmental Information Processing; Signal Transduction; Two-component system; K02483
K02519	0.00	0.00	125.00	181.50	249.50	Genetic Information Processing; Translation; Translation factors; K02519
K03043	0.00	0.00	200.00	289.00	362.50	Metabolism; Nucleotide Metabolism; Purine/Pyrimidine metabolism; K03043
K03046	0.00	0.00	194.00	310.00	352.00	Metabolism; Nucleotide Metabolism; Purine/Pyrimidine metabolism; K03046

Supplementary Table 8 (cont.) Summary of KEGG Orthology

KEGG ORTHOLOGY	*P*	FDR-corrected *P*	Norman	Matses	Tunapuco	Function
K03070	0.00	0.00	166.00	246.00	256.00	Genetic Information Processing; Folding, Sorting and Degradation; Protein export; K03070
K03088	0.00	0.00	551.00	351.00	272.50	Genetic Information Processing; Transcription; Transcription machinery; K03088
K03091	0.00	0.01	249.00	237.50	131.00	Genetic Information Processing; Transcription; Transcription machinery; K03091
K03168	0.00	0.00	130.00	184.00	218.00	Genetic Information Processing; Replication and Repair; DNA replication proteins; K03168
K03169	0.00	0.00	319.00	288.50	197.50	Genetic Information Processing; Replication and Repair; DNA replication proteins; K03169
K03205	0.00	0.00	543.00	347.50	157.00	Environmental Information Processing; Membrane Transport; Bacterial secretion system; K03205
K03296	0.00	0.00	309.00	192.00	280.50	Unclassified; Cellular Processes and Signaling; Other ion-coupled transporters; K03296
K03315	0.00	0.00	136.00	153.00	214.00	Metabolism; Energy Metabolism; Methane metabolism; K03315
K03423	0.00	0.01	167.00	204.00	213.00	Unclassified; Metabolism; Others; K03423
K03427	0.00	0.00	229.00	403.00	373.50	Unclassified; Genetic Information Processing; Restriction enzyme; K03427
K03495	0.00	0.00	128.00	198.50	201.50	Genetic Information Processing; Replication and Repair; Chromosome; K03495
K03496	0.00	0.01	281.00	297.00	238.50	Cellular Processes; Cell Motility; Cytoskeleton proteins; K03496
K03497	0.00	0.00	327.00	291.50	222.00	Genetic Information Processing; Transcription; Transcription factors; K03497
K03555	0.00	0.00	153.00	196.50	263.00	Genetic Information Processing; Replication and Repair; DNA repair and recombination proteins; K03555
K03654	0.00	0.00	132.00	165.50	280.50	Genetic Information Processing; Replication and Repair; DNA repair and recombination proteins; K03654
K03655	0.00	0.00	267.00	446.50	542.50	Genetic Information Processing; Replication and Repair; DNA repair and recombination proteins; K03655
K03695	0.00	0.00	138.00	188.00	212.50	Genetic Information Processing; Folding, Sorting and Degradation; Chaperones and folding catalysts; K03695
K03701	0.00	0.00	222.00	385.50	471.00	Genetic Information Processing; Replication and Repair; DNA repair and recombination proteins; K03701
K03702	0.00	0.00	123.00	216.00	214.00	Genetic Information Processing; Replication and Repair; DNA repair and recombination proteins; K03702
K03723	0.00	0.00	154.00	198.50	273.00	Genetic Information Processing; Replication and Repair; DNA repair and recombination proteins; K03723
K03737	0.00	0.00	181.00	224.50	308.00	Metabolism; Energy Metabolism; Carbon fixation pathways in prokaryotes; K03737
K04043	0.00	0.00	141.00	225.00	217.50	Genetic Information Processing; Folding, Sorting and Degradation; RNA degradation; K04043
K04759	0.00	0.01	253.00	318.50	331.00	Unclassified; Cellular Processes and Signaling; Other transporters; K04759
K05349	0.00	0.00	278.00	256.00	407.00	Metabolism; Carbohydrate Metabolism; Starch and sucrose metabolism; K05349
K05366	0.00	0.00	130.00	180.00	249.00	Metabolism; Glycan Biosynthesis and Metabolism; Glycosyltransferases; K05366
K06147	0.01	0.02	1034.00	1073.00	858.00	Environmental Information Processing; Membrane Transport; Transporters; K06147
K06148	0.00	0.01	244.00	333.50	227.50	Environmental Information Processing; Membrane Transport; ABC transporters; K06148
K06180	0.00	0.00	158.00	199.00	208.50	Genetic Information Processing; Translation; Ribosome Biogenesis; K06180
K06400	0.00	0.00	649.00	465.00	286.50	Unclassified; Cellular Processes and Signaling; Sporulation; K06400
K06919	0.00	0.00	295.00	172.00	108.50	Unclassified; Genetic Information Processing; Replication, recombination and repair proteins; K06919
K06921	0.00	0.00	79.00	180.50	203.00	Unclassified; Poorly Characterized; General function prediction only; K06921
K06950	0.00	0.00	169.00	210.00	213.00	Unclassified; Poorly Characterized; General function prediction only; K06950

Supplementary Table 8 (cont.) Summary of KEGG Orthology						
**KEGG ORTHOLOGY**	***P***	**FDR-corrected *P***	**Norman**	**Matses**	**Tunapuco**	**Function**
K07030	0.00	0.00	220.00	170.50	148.50	Unclassified; Poorly Characterized; General function prediction only; K07030
K07114	0.01	0.03	459.00	277.00	465.00	Unclassified; Poorly Characterized; General function prediction only; K07114
K07133	0.00	0.00	297.00	924.50	1293.50	Unclassified; Poorly Characterized; General function prediction only; K07133
K07137	0.00	0.00	155.00	206.00	174.50	Unclassified; Poorly Characterized; General function prediction only; K07137
K07319	0.00	0.00	119.00	191.50	225.50	Unclassified; Genetic Information Processing; Replication, recombination and repair proteins; K07319
K07495	0.00	0.00	24.00	104.50	305.50	Unclassified; Genetic Information Processing; Replication, recombination and repair proteins; K07495
K07497	0.00	0.01	243.00	338.00	375.50	Unclassified; Genetic Information Processing; Replication, recombination and repair proteins; K07497
K07636	0.00	0.00	237.00	165.00	171.50	Metabolism; Enzyme Families; Protein kinases; K07636
K07658	0.00	0.00	237.00	161.00	119.00	Environmental Information Processing; Signal Transduction; Two-component system; K07658
K07720	0.00	0.00	358.00	202.00	213.00	Environmental Information Processing; Signal Transduction; Two-component system; K07720
K08303	0.00	0.00	171.00	207.50	249.00	Metabolism; Enzyme Families; Peptidases; K08303
K09686	0.00	0.00	221.00	157.50	141.00	Environmental Information Processing; Membrane Transport; ABC transporters; K09686
K09687	0.00	0.00	296.00	210.50	147.50	Environmental Information Processing; Membrane Transport; ABC transporters; K09687
K09691	0.01	0.02	373.00	293.50	241.00	Environmental Information Processing; Membrane Transport; ABC transporters; K09691
K09810	0.00	0.00	575.00	512.50	384.50	Environmental Information Processing; Membrane Transport; Transporters; K09810
K12373	0.00	0.00	236.00	117.00	217.00	Metabolism; Carbohydrate Metabolism; Amino sugar and nucleotide sugar metabolism; K12373

Supplementary Table 9. Summary of Enzyme Commission (EC) codes with significantly different relative abundances (Kruskal-Wallis, FDR-adjusted *P* < 0.05) among the three study populations (see attached Excel sheet).

EC code	*P*	FDR-corrected *P*	Norman	Matses	Tunapuco	Function
1.1.1.205	0.00	0.00	119.50	157.25	185.25	IMP Dehydrogenase
1.1.1.22	0.00	0.00	92.50	140.50	127.50	UDP-glucose dehydrogenase
1.1.1.274	0.00	0.00	32.50	73.75	30.00	didehydrogluconate reductase
1.1.1.40	0.00	0.00	20.50	32.50	59.75	Malate dehydrogenase
1.17.7.1	0.00	0.00	87.50	125.25	142.00	diphosphate synthase
1.3.99.1	0.00	0.00	164.50	230.50	270.25	Succinate dehydrogenase
1.4.1.14	0.00	0.00	335.50	482.25	595.25	Glutamate synthase
1.8.1.8	0.00	0.00	25.00	33.75	71.00	Protein disulfide reductase
2.1.1.14	0.00	0.00	58.00	112.75	91.25	Homocysteine methylase
2.1.1.31	0.00	0.00	75.00	114.75	102.25	
2.1.1.72	0.00	0.00	496.50	838.00	757.00	DNA methytransferase
2.1.2.1	0.00	0.00	88.50	143.50	145.00	Glycine hydroxymethyltransferase
2.1.2.3	0.00	0.00	99.50	144.75	162.50	
2.2.1.7	0.00	0.00	134.00	188.00	245.50	Xylulose-5-phosphate synthase
2.4.1.11	0.00	0.00	16.50	29.25	62.50	Glycogen synthase
2.4.1.182	0.00	0.00	20.50	28.50	64.00	Lipid-A-disaccharide syntahse
2.4.1.25	0.00	0.00	164.00	219.75	301.50	4-alpha-glucanotransferase
2.4.2.7	0.00	0.00	54.50	89.00	69.00	Adenine phosphoribosyltransferase
2.5.1.17	0.00	0.00	58.00	25.00	25.00	Cob(I)yrinic acid a,c-diamide adenosyltransferase
2.5.1.75	0.00	0.00	69.00	94.75	105.25	tRNA dimethylallyltransferase
2.6.1.1	0.00	0.00	106.00	144.75	197.25	Aspartate transaminase
2.7.1.45	0.00	0.00	82.00	129.75	112.00	2-keto-3-deoxygluconokinase
2.7.1.48	0.00	0.00	108.00	154.00	182.25	Uridine kinase
2.7.1.6	0.00	0.00	89.50	129.75	136.50	Galactokinase
2.7.1.90	0.00	0.00	15.50	45.00	58.50	Fructose-6-phosphate phosphotransferase
2.7.13.3	0.00	0.00	934.00	596.50	623.75	Histidine kinase
2.7.6.2	0.00	0.00	51.50	31.75	30.50	Thiamine diphosphokinase
2.7.7.19	0.00	0.00	26.00	35.50	85.00	Polynucleotide adenylyltransferase
2.7.7.24	0.00	0.00	72.00	128.25	122.25	Glucose-1-phosphate thymidylyltransferase
2.7.7.6	0.00	0.00	540.00	790.75	930.25	DNA -directed RNA polymerase
2.7.7.8	0.00	0.00	129.50	182.00	198.75	Polynucleotide phosphorylase
2.7.9.2	0.00	0.00	32.50	62.50	117.00	Phophoenolpyruvate synthase
3.1.21.3	0.00	0.00	380.00	671.75	721.75	DNase Type I
3.1.21.4	0.00	0.00	74.50	114.75	147.50	DNase Type II
3.1.21.5	0.00	0.00	32.00	67.75	76.50	DNase Type III
3.1.3.48	0.00	0.00	175.00	122.00	111.00	Phospho-tyrosine-phosphatase
3.2.1.1	0.00	0.00	98.50	156.25	244.00	Alpha-amylase
3.2.1.40	0.00	0.00	102.50	56.25	82.25	Alpha-L-rhamnosidase
3.2.1.52	0.00	0.00	288.50	159.25	242.50	Hexosaminidase
3.4.11.4	0.00	0.00	61.00	80.75	117.25	Aminopeptidase
3.4.11.9	0.00	0.00	110.00	133.25	166.75	Xaa-Pro aminopeptidase
3.4.14.4	0.00	0.00	28.50	46.75	97.25	Dipeptidyl-peptidase III
3.4.21.53	0.00	0.00	162.50	195.75	253.00	ATP-dependent serine proteinase
3.4.22.40	0.00	0.00	41.00	67.75	139.50	Aminopeptidase C
3.5.1.53	0.00	0.00	28.50	41.50	59.75	N-carbamoylputrescine amidase
3.5.4.12	0.00	0.00	70.00	107.00	83.00	dCMP deaminase
3.5.99.6	0.00	0.00	88.00	102.50	146.75	Glucosamine-6-phosphate deaminase
3.6.3.12	0.00	0.00	89.00	4.50	4.25	Potassium-transporting ATPase
3.6.3.2	0.00	0.00	28.50	142.00	22.75	Magnesium-translocating ATPase
3.6.4.12	0.00	0.00	1024.50	1324.25	1641.75	DNA helicase
3.6.4.13	0.00	0.00	55.50	87.75	146.50	RNA helicase
4.1.1.19	0.00	0.00	65.00	87.25	116.25	Arginine decarboxylase
4.1.1.49	0.00	0.00	75.00	102.25	122.75	Phosphoenolpyruvate carboxykinase
4.1.2.4	0.00	0.00	60.50	98.00	69.50	Deoxyribose-phosphate aldolase
4.1.3.6	0.00	0.00	25.50	83.50	68.00	Citrate lyase
4.2.1.3	0.00	0.00	127.00	188.25	215.50	Aconitate hydatase
4.2.1.46	0.00	0.00	138.00	183.25	213.25	dTDP-glucose dehydratase
4.3.2.2	0.00	0.00	99.50	145.75	167.75	Adenylosuccinate lyase
4.4.1.16	0.00	0.00	81.00	114.00	124.75	Selenocysteine lyase

Supplementary Table 9 (cont.) Summary of ECs

EC code	*P*	FDR-corrected *P*	Norman	Matses	Tunapuco	Function
4.4.1.5	0.00	0.00	67.00	48.00	27.50	Lactoylglutathione lyase
4.99.1.3	0.00	0.00	72.50	48.25	35.25	Cobaltochelatase
5.1.3.14	0.00	0.00	75.00	121.75	98.50	UDP-N-acetylglucosamine 2-epimerase
5.3.1.13	0.00	0.00	17.00	34.25	55.50	Arabinose-5-phosphate isomerase
5.4.2.6	0.00	0.00	68.50	32.25	31.50	Beta-phophoglucomutase
5.4.2.8	0.00	0.00	130.00	180.75	197.25	Phosphomannomutase
6.1.1.12	0.00	0.00	137.50	210.75	211.25	Aspartate tRNA ligase
6.1.1.16	0.00	0.00	102.50	161.25	158.50	Cysteine tRNA ligase
6.1.1.17	0.00	0.00	108.00	148.25	165.00	Glutamate tRNA ligase
6.1.1.19	0.00	0.00	109.50	161.00	199.00	Arginine tRNA ligase
6.1.1.20	0.00	0.00	215.00	275.50	354.50	Phenylalanine tRNA ligase
6.1.1.22	0.00	0.00	84.50	129.25	126.50	Asparagine tRNA ligase
6.1.1.4	0.00	0.00	151.50	236.25	252.25	Leucine tRNA ligase
6.1.1.7	0.00	0.00	139.50	179.25	231.75	Alanine tRNA ligase
6.1.1.9	0.00	0.00	150.50	225.00	227.00	Valine tRNA ligase
6.2.1.1	0.00	0.00	42.00	73.75	117.00	Acetate CoA ligase
6.2.1.3	0.00	0.00	109.00	156.75	234.50	Fatty acid CoA ligase
6.3.2.9	0.00	0.00	86.00	103.25	129.00	D-glutamate ligase
6.3.4.2	0.00	0.00	109.50	164.50	181.00	CTP synthase
6.3.5.1	0.00	0.00	97.50	114.50	146.25	NAD+ synthase
6.3.5.10	0.00	0.00	63.50	42.25	37.50	Adenosylcobyric acid synthase
6.3.5.3	0.00	0.00	167.50	223.00	294.25	Phosphoribosylformulglycinamidine synthase
1.6.-.-	0.00	0.00	71.00	17.50	18.50	
1.6.5.-	0.00	0.00	71.50	139.25	274.75	
2.-.-.-	0.00	0.00	59.50	90.50	117.25	
3.4.-.-	0.00	0.00	382.00	485.25	615.50	
3.4.21.-	0.00	0.00	77.00	112.75	214.75	
3.4.24.-	0.00	0.00	384.50	463.75	595.25	
3.6.1.-	0.00	0.00	471.50	594.00	741.00	
3.6.3.-	0.00	0.00	187.00	268.50	331.00	
5.-.-.-	0.00	0.00	131.00	202.50	227.00	
5.99.1.-	0.00	0.00	71.50	157.50	235.50	

Supplementary Table 10. Prevalence of Spirochaetes in human gut microbiomes studied to date. A minimum of 0.01% relative abundance was used to denote presence of the taxon.

Population	Individuals positive for Spirochaetes (n)	Total individuals analyzed (n)	Max. observed frequency (%)	Mean frequency (%, mean ± s.d)
Venezuela[1]	28	88	8.25	0.47±1.28
Malawi[1]	21	59	13.72	0.78±2.22
Tunapuco[b]	29	31	42.01	7.17±9.79
Hadza[2]	27	27	21.31	2.85±4.43
Matses[b]	18	25	60.78	10.69±15.38
USA (St. Louis, Philadelphia) [1]	0	85	<0.01	<0.001
USA (St. Louis, Houston)[a]	5	182	0.18	<0.001
USA (Norman) [b]	8	23	0.03	<0.005
Italy[2]	3	16	0.02	<0.001
Notes:<br>[a] obtained from the HMP consortium<br>[b] generated in this study				

Supplementary Table 11. Prevalence of *Treponema* OTUs in traditional human gut microbiomes shown in Supplementary Figure 4.

OTU-ID	338950	4307383	300310	1145921	514045
Venezuela	0	9	2	1	0
Malawi	3	4	11	5	0
Matses	12	2	16	0	0
Tunapuco	16	16	21	9	0
Hadza	8	5	0	5	3

Supplementary Table 12. Summary of assembly and annotation statistics for shotgun metagenomic datasets from the three study populations.

Sample	Population	Total Number of Contigs	Total Assembled Length(Mb)	Max Length (Kb)	N50 (bp)	No: of Predicted ORFs	No: of ORFs assigned annotation
HCO02	Tunapuco	404,654	71.96	27.21	162	242,193	106,505
HCO07	Tunapuco	510,737	97.57	80.52	172	305,981	112,428
HCO09	Tunapuco	371,470	81.57	93.79	217	243,912	132,622
HCO11	Tunapuco	396,800	95.98	155.86	245	280,276	111,390
HCO12	Tunapuco	445,200	93.37	84.49	202	292,281	92,773
HCO53	Tunapuco	357,156	77.03	281.85	204	232,696	67,351
HCO61	Tunapuco	486,418	105.99	148.14	206	320,571	98,294
HCO64	Tunapuco	484,137	103.40	135.72	200	309,719	144,838
HCO67	Tunapuco	529,272	107.06	182.78	187	341,395	107,386
HCO70	Tunapuco	633,315	141.06	221.64	215	427,081	177,979
HCO72	Tunapuco	457,846	91.16	191.63	181	286,465	132,340
HCO74	Tunapuco	605,770	115.77	331.31	169	361,216	156,556
NO01	Norman	195,335	96.19	291.71	2306	185,241	135,274
NO02	Norman	726,378	234.80	335.14	490	561,246	301,277
NO03	Norman	713,512	217.19	242.93	407	534,657	304,896
NO04	Norman	679,179	219.57	203.80	474	530,098	327,599
NO05	Norman	635,076	204.68	403.22	464	490,585	308,482
NO06	Norman	552,294	166.77	292.33	390	420,798	285,989
NO08	Norman	405,452	136.30	440.87	569	318,936	196,124
NO09	Norman	289,719	116.95	166.73	1063	246,193	167,415
NO10	Norman	461,035	161.85	291.61	597	373,220	237,805
NO11	Norman	569,479	200.73	335.61	570	470,968	295,314
NO12	Norman	341,661	145.00	385.03	885	303,670	214,857
NO13	Norman	759,031	236.35	312.54	447	573,248	286,617
NO14	Norman	438,032	159.93	309.86	724	358,553	236,955
NO15	Norman	719,845	183.60	242.90	283	495,897	269,184
NO16	Norman	561,215	198.61	290.44	596	455,184	298,938
NO17	Norman	366,298	138.78	370.56	773	306,925	184,304
NO18	Norman	367,559	130.62	404.07	703	293,604	178,013
NO19	Norman	506,152	181.32	342.97	757	403,950	219,860

Supplementary Table 12 (cont.) Summary of assembly and annotation statistics							
Sample	Population	Total Number of Contigs	Total Assembled Length(Mb)	Max Length (Kb)	N50 (bp)	No: of Predicted ORFs	No: of ORFs assigned annotation
NO20	Norman	360,768	148.47	241.60	957	314,569	228,984
NO21	Norman	624,887	193.12	368.32	414	476,167	305,188
NO22	Norman	597,606	189.57	346.73	455	458,080	267,487
NO23	Norman	572,926	176.11	427.24	422	429,930	253,445
SM01	Matses	561,652	161.82	298.14	391	386,774	167,631
SM02	Matses	861,183	247.02	218.27	381	598,049	208,211
SM03	Matses	563,294	141.88	280.12	282	373,288	153,391
SM05	Matses	171,615	79.91	206.33	2479	150,206	108,096
SM11	Matses	591,314	168.56	319.37	364	409,275	141,532
SM18	Matses	631,714	173.19	327.90	351	425,220	169,337
SM20	Matses	551,078	137.72	318.80	273	367,662	121,729
SM23	Matses	457,367	136.88	397.45	427	321,310	125,679
SM24	Matses	258,885	91.84	313.12	667	199,645	124,416
SM25	Matses	395,693	141.83	241.74	682	310,408	145,983
SM28	Matses	529,597	132.46	213.05	275	362,426	138,026
SM29	Matses	760,972	187.63	227.72	272	497,800	160,171
SM30	Matses	597,215	171.17	183.06	361	424,930	192,656
SM31	Matses	642,808	155.81	234.60	261	404,222	142,913
SM32	Matses	586,577	162.59	348.43	347	398,409	118,951
SM33	Matses	597,589	157.32	263.49	320	396,365	110,563
SM34	Matses	640,020	173.97	326.77	323	439,892	194,473
SM37	Matses	652,611	170.36	192.79	314	434,043	169,444
SM39	Matses	676,159	186.18	285.59	351	461,872	170,336
SM40	Matses	676,822	182.38	227.43	332	447,456	135,523
SM41	Matses	475,684	157.21	149.81	650	344,001	151,121
SM42	Matses	612,538	157.78	337.96	295	403,194	153,405
SM43	Matses	516,926	151.37	213.54	380	367,178	173,944
SM44	Matses	540,623	146.99	222.39	334	362,640	144,205

Supplementary Table 13. Assembly statistics for partial genomes of gut *Treponema* strains reconstructed from Matses shotgun metagenomic data.

***Treponema* Strain**	**Strain 1**				**Strain 2**	
**Sample**	SM03	SM23	SM28	SM42	SM23	SM42
Total length	~2.46Mb	~2.3Mb	~2.22Mb	~2.19Mb	~2.43Mb	~2.37Mb
Number of contigs	66	50	49	156	51	29
Length in contigs with marker loci	861Kb	1.28Mb	1.15Mb	449Kb	1Mb	1.34Mb
Number of contigs with marker loci	11	11	11	16	13	11
Depth of Coverage	~97	~135	~513	~26	~58	~331
FCD errors	2	3	4	3	1	5
Regions of low coverage	4	1	0	3952	190	0
% error free bases	~95%	~96%	~92%	~73%	~87%	~97%
Number of predicted ORFs	2250	2087	1983	1970	2073	2068
Number of ORFs with function assigned	1570	1498	1462	1428	1475	1480

SUPPLEMENTARY REFERENCES

1. Schnorr SL, et al. Gut microbiome of the Hadza hunter-gatherers. *Nature communications* 5, (2014).
2. Yatsunenko T, et al. Human gut microbiome viewed across age and geography. *Nature* 486, 222-227 (2012).
